# Serological evidence of *Leishmania* spp. exposure in rabbits and hares: A systematic review and meta-analysis

**DOI:** 10.1016/j.crpvbd.2026.100453

**Published:** 2026-10-27

**Authors:** Ali Asghari, Giovanni Sgroi, Zaid Hassan Mohsin Alsafi, Laya Shamsi

**Affiliations:** a Medical Microbiology Research Center, Qazvin University of Medical Sciences, Qazvin, Iran; b Department of Sciences and Technologies, University of Sannio, Benevento, Italy; c Experimental Zooprophylactic Institute of Southern Italy, Portici, (Naples), Italy; d College of Veterinary Medicine, University of Wasit, Al Kut, Wasit, Iraq; e Department of Veterinary Pathobiology, SR.C., Islamic Azad University, Tehran, Iran

**Keywords:** *Leishmania*, Lagomorphs, Rabbits, Hares, Seroprevalence, Meta-analysis

## Abstract

Rabbits and hares are increasingly recognized as potentially relevant hosts in the epidemiology of *Leishmania* spp., yet the serological evidence across available studies remains fragmented and highly variable. This systematic review and meta-analysis aimed to quantitatively synthesize the published evidence on anti-*Leishmania* spp. seropositivity in rabbits and hares and to investigate geographical, host-related, temporal, and methodological sources of variation. PubMed, Scopus, and Web of Science were systematically searched for studies published between 1 January 2000 and 1 August 2026, supplemented by Google Scholar and reference list screening. Eligible studies reporting animal-level serological data for rabbits or hares were included. Pooled seroprevalence was estimated using a random-effects model with logit-transformed event rates. Heterogeneity, subgroup analyses, univariable meta-regression, leave-one-out and sample-size sensitivity analyses, and publication-bias assessments were performed. Host-level associations with age and sex were evaluated using pooled odds ratios (ORs). Eighteen studies contributed 29 eligible serological datasets. Based on the available published evidence, the pooled seroprevalence of anti-*Leishmania* spp. antibodies in lagomorphs was 17.3% (95% CI: 12.0–24.2%), with substantial between-dataset heterogeneity (*I*^2^ = 95.0%). The estimate remained stable in leave-one-out analyses (16.0–18.7%) and after excluding the dataset with < 10 animals (17.4%, 95% CI: 12.0–24.4%). Significant differences were observed by country (*P* < 0.001), antibody endpoint (*P* < 0.001), and hare species (*P* = 0.008). In contrast, differences according to host type, publication period, sample size, serological method, and rabbit husbandry type were not statistically significant. Meta-regression showed no significant association with publication year (*P* = 0.067) or sample size (*P* = 0.286). Neither age (OR = 1.69, 95% CI: 0.92–3.10) nor sex (OR = 1.00, 95% CI: 0.68–1.47) was significantly associated with seropositivity. Egger’s test showed no significant funnel-plot asymmetry (*P* = 0.090), and trim-and-fill analysis imputed no missing studies. The available evidence indicates substantial but highly heterogeneous anti-*Leishmania* spp. seropositivity in rabbits and hares, with significant variation by country, antibody endpoint, and hare species. These findings support the epidemiological relevance of lagomorph exposure while highlighting the need for more standardized and geographically diverse serological studies.

## Highlights

• A systematic review and meta-analysis including 18 studies comprising 29 serological datasets published during 2000–2026.
• Overall anti-*Leishmania* spp. seroprevalence in lagomorphs was 17.3%.
• Pooled seroprevalence was 18.6% in rabbits and 12.4% in hares.
• Pooled seroprevalence was 20.5% and 10.7% in sylvatic and domestic rabbits, respectively.
• Seroprevalence varied significantly by country, antibody endpoint, and hare species.

## Graphical abstract

**Figure:**
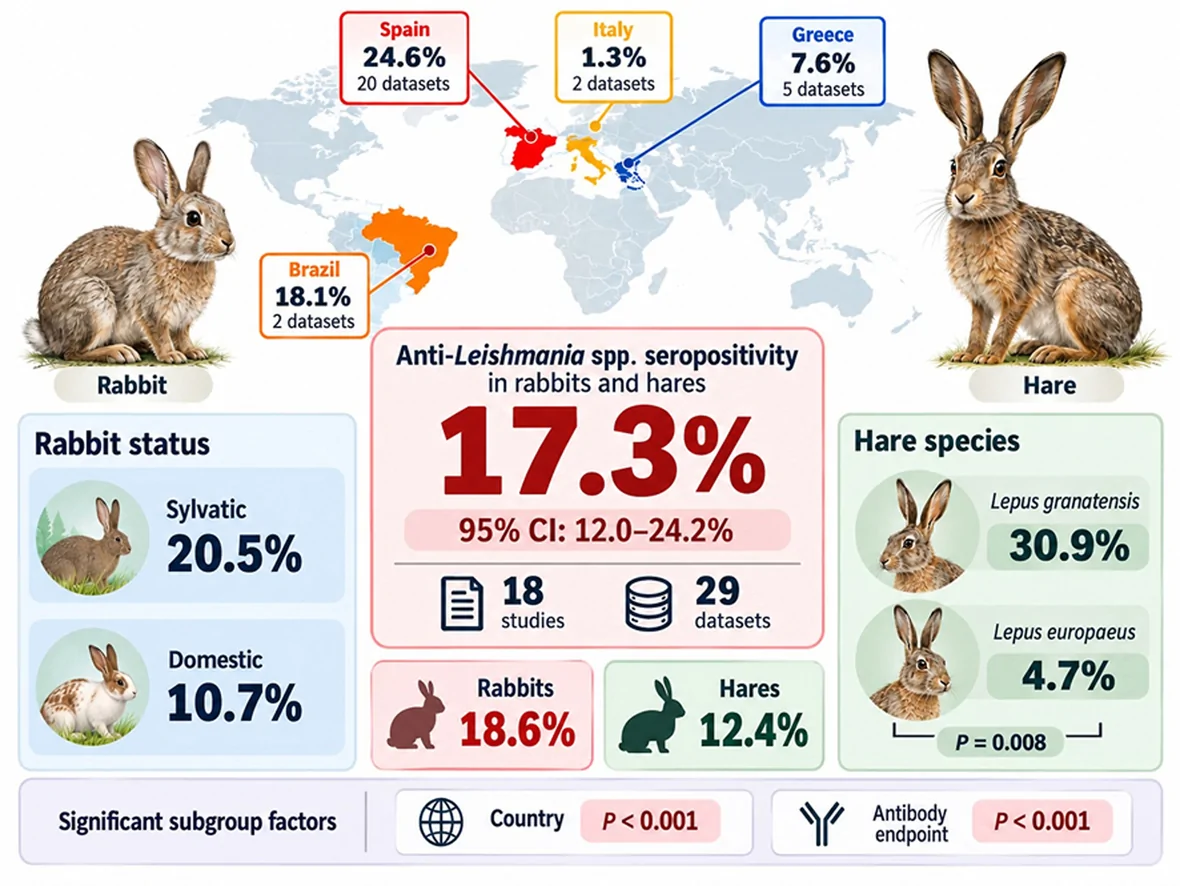

## 1 Introduction

Leishmaniasis comprises a group of vector-borne diseases caused by protozoan parasites of the genus *Leishmania* and transmitted primarily by phlebotomine sand flies (Asghari et al., 2022; Cecílio et al., 2022). Its transmission is maintained through complex interactions among vectors, vertebrate hosts, and environmental conditions, and the relative importance of different animal hosts varies considerably among epidemiological settings (Serafim et al., 2021; Tsakmakidis et al., 2024). In Mediterranean regions, domestic dogs are recognized as the principal reservoir of *Leishmania infantum*; however, increasing evidence indicates that sylvatic hosts may also contribute to parasite maintenance, particularly where abundant susceptible hosts coexist with competent sand fly vectors (Azami-Conesa et al., 2021; Shams et al., 2022; Tsakmakidis et al., 2024).

Among sylvatic mammals, lagomorphs have received increasing epidemiological attention. Evidence from Spain has shown that Iberian hares (*Lepus granatensis*) can infect *Phlebotomus perniciosus* through xenodiagnosis, supporting their potential reservoir competence (Molina et al., 2012). Sylvatic rabbits (*Oryctolagus cuniculus*) have also been implicated in *L. infantum* transmission, with recent investigations demonstrating high levels of parasite detection and epidemiological links between infected rabbit populations and areas of human transmission (Martín-Sánchez et al., 2024). A large-scale survey conducted in Spanish Mediterranean ecosystems further demonstrated widespread but heterogeneous exposure to *L. infantum* in sylvatic rabbits and Iberian hares (Barbero-Moyano et al., 2024). More recently, long-term surveillance of sylvatic rabbits sampled between 2009 and 2023 showed persistent serological evidence of exposure and marked spatial variation over time, highlighting the potential importance of lagomorph populations in maintaining local transmission cycles (Barbero-Moyano et al., 2026). Serological evidence has also been reported in domestic rabbits outside the Mediterranean region, including recent findings from northeastern Brazil (Silva et al., 2024).

Serology represents an important approach for assessing previous or ongoing exposure to *Leishmania* spp. in animal populations, particularly in epidemiological surveys in which direct parasite detection may be limited by tissue availability or parasite distribution (Paltrinieri et al., 2016; Morales-Yuste et al., 2022). However, substantial methodological diversity exists among lagomorph studies. Indirect fluorescent antibody test (IFAT), enzyme-linked immunosorbent assay (ELISA), direct agglutination test (DAT), and immunochromatographic tests (ICTs) have been used with different antigens, antibody endpoints, and positivity thresholds (de la Cruz et al., 2016; Tsakmakidis et al., 2019). Diagnostic performance may also vary according to the assay and cut-off selected, and inter-laboratory variation in serological testing has been demonstrated in veterinary *Leishmania* spp. diagnostics (de la Cruz et al., 2016; Mahachi et al., 2024). Consequently, detection of anti-*Leishmania* spp. antibodies should primarily be interpreted as evidence of host exposure and does not, by itself, establish active infection or reservoir competence.

Despite the growing number of seroepidemiological investigations, evidence regarding *Leishmania* spp. exposure in rabbits and hares remains fragmented across geographical areas, host populations, diagnostic approaches, and study periods. Previous reviews have highlighted the diversity of sylvatic animal species exposed to or infected with zoonotic *Leishmania* spp., but a dedicated quantitative synthesis of serological evidence in lagomorphs remains lacking (Azami-Conesa et al., 2021). Therefore, this systematic review and meta-analysis aimed to estimate the pooled seroprevalence of anti-*Leishmania* spp. antibodies in rabbits and hares based on the available published evidence and to investigate potential geographical, host-related, temporal, and methodological sources of heterogeneity through subgroup analyses and meta-regression. Where sufficient data were available, associations of seropositivity with host age and sex were also quantitatively evaluated.

## 2 Materials and methods

### 2.1 Study design and reporting

This systematic review and meta-analysis were conducted to synthesize the available published serological evidence of exposure to *Leishmania* spp. in rabbits and hares. The review process was structured in accordance with the Preferred Reporting Items for Systematic Reviews and Meta-Analyses (PRISMA) recommendations (Page et al., 2021). Because serological positivity indicates an antibody response to *Leishmania* spp. exposure but does not necessarily demonstrate active infection or reservoir competence, the primary outcome was defined as the animal-level seroprevalence of anti-*Leishmania* spp. antibodies. Pooled estimates were interpreted as quantitative summaries of the available published evidence rather than as estimates of worldwide seroprevalence.

### 2.2 Literature search

A systematic literature search was conducted in PubMed, Scopus, and Web of Science for studies published from 1 January 2000 to 1 August 2026. The search strategy combined terms related to *Leishmania* spp. with terms representing lagomorph hosts, including “rabbits”, “hares”, “lagomorphs”, “leporids”, “*Oryctolagus*”, and “*Lepus*”. The searches were designed to identify relevant studies irrespective of the diagnostic approach used, after which studies containing eligible serological data were selected for the present analysis. To improve retrieval sensitivity, Google Scholar was used as a supplementary manual search source rather than as a systematically searched database. Searches were performed using combinations of the same keywords used in the primary database searches, namely “*Leishmania*”, “leishmaniasis”, “*Leishmania infantum*”, “rabbit”, “rabbits”, “hare”, “hares”, “lagomorph”, “lagomorphs”, “leporid”, “leporids”, “*Oryctolagus*”, “*Oryctolagus cuniculus*”, “*Lepus*”, “*Lepus europaeus*”, and “*Lepus granatensis*”, and the first 50 pages of Google Scholar results were screened. For records not already identified through PubMed, Scopus, or Web of Science, titles and abstracts were assessed for potential eligibility, and potentially relevant articles subsequently underwent the same full-text screening and eligibility assessment as records retrieved from the primary databases. In addition, the reference lists of relevant original articles and review papers were manually examined to identify potentially eligible studies not captured by the database searches. Duplicate records were removed before screening. Titles and abstracts were initially assessed for relevance, followed by full-text evaluation against the predefined eligibility criteria. The complete study-selection process was documented using a PRISMA flow diagram.

### 2.3 Eligibility criteria and study selection

Original studies were considered eligible when they investigated rabbits (*Oryctolagus cuniculus*) and/or hares (*Lepus* spp.). Among the included studies, the hare species represented were *Lepus europaeus* and *Lepus granatensis*. Eligible studies were required to assess anti-*Leishmania* spp. antibodies using a serological method and to report an animal-level denominator together with the number of seropositive animals or provide sufficient information for these values to be derived. Studies involving other lagomorphs or animal species without separately extractable rabbit or hare data were excluded. Reports lacking a useable denominator, studies based solely on selected positive or pathological subsets that could not provide a representative seroprevalence estimate, and dependent duplicate outcomes from the same animals were not entered independently into the primary meta-analysis. A single article could contribute more than one dataset when separate, independently interpretable populations were reported, such as distinct host species, non-overlapping sampling periods, or other clearly separable animal groups. Conversely, results arising from the same animals were not treated as statistically independent simply because different assays, antibody thresholds, or sample types had been applied. When a study reported combined rabbit-and-hare results without species-specific positive counts, the dataset was retained for the overall lagomorph analysis but excluded from host-specific comparisons. Potential population overlap among several studies conducted in the Madrid transmission focus in Spain (García et al., 2014; Jiménez et al., 2014; Moreno et al., 2014; de la Cruz et al., 2016; Ortega et al., 2017) could not always be resolved with certainty; these datasets were retained because definitive duplication could not be established, and this potential non-independence was considered when interpreting the findings.

### 2.4 Data extraction and definition of seropositivity

Data were extracted into a standardized database and included first author and publication year, sampling period, country, host type, rabbit husbandry status where applicable, number of animals examined, number of seropositive animals, serological method, antibody endpoint, positivity threshold, and available sex- and age-specific results. The extraction database also retained detailed study-specific information on the interpretation of serological cut-offs and doubtful or inconclusive results. The serological methods represented in the eligible literature were the IFAT, ELISA, DAT, and ICTs. Antibody endpoints were classified as IgG, total or undifferentiated antibodies, or unspecified antibody class according to the information provided by the original study. A conservative outcome-definition rule was applied throughout the primary meta-analysis. When multiple titres or positivity thresholds were reported, only animals meeting the more definitive positive criterion were counted as seropositive. Animals categorized as doubtful, borderline, inconclusive, or reactive only at a lower non-definitive threshold were not included in the positive numerator. Lower or more permissive cut-offs were not substituted for the locked primary outcome and were considered only as potential sensitivity information where appropriate. This approach was adopted to reduce the risk of artificially inflating pooled seroprevalence because serological thresholds varied substantially among studies.

### 2.5 Methodological quality assessment

The methodological quality of the included studies was assessed using an eight-item Joanna Briggs Institute (JBI)-based critical appraisal framework (Lin et al., 2025). The assessment considered whether the criteria for inclusion in the sample were clearly defined, whether the study subjects and setting were adequately described, whether exposure was measured validly and reliably, whether objective and standard criteria were used to measure the condition, whether potential confounding factors (e.g. host age, sex, species, host status or husbandry, geographical location, and sampling period or season) were identified, whether strategies for addressing confounding were reported, whether outcomes were measured validly and reliably, and whether appropriate statistical analyses were used. Each item was scored as 1 for “Yes”, 0.5 for “Unclear”, and 0 for “No” producing a maximum possible score of 8. Studies with total scores of ≤ 3.5, 4.0–6.0, and 6.5–8.0 were classified as low-, moderate-, and high-quality studies, respectively. The quality assessment was used to characterize the strength and methodological limitations of the available evidence.

### 2.6 Primary meta-analysis and assessment of heterogeneity

All quantitative analyses were performed using Comprehensive Meta-Analysis version 3 (CMA; Biostat, Englewood, NJ, USA) (Mahdavi et al., 2024). Animal-level event rates were entered as the number of seropositive animals and the corresponding total number examined. Event rates were analyzed on the logit scale and subsequently back-transformed to proportions for presentation as pooled seroprevalence percentages with 95% confidence intervals (CIs). Because substantial clinical, geographical, and methodological diversity was anticipated across the included studies, the random-effects model was used as the primary analytical approach (Mamizadeh et al., 2025). Fixed-effect estimates generated by the software were not used as the principal pooled results. For zero-event datasets, a continuity correction of 0.5 was applied for calculation of the logit event rate and its variance, allowing such datasets to remain in the analysis rather than being excluded solely because no seropositive animals were detected. Statistical heterogeneity was evaluated using Cochran’s *Q* statistic, its associated *P*-value, the *I*^2^ statistic, and the between-study variance estimates *τ*^2^ and *τ*. *I*^2^ values > 75% were interpreted as indicating substantial heterogeneity. Statistical tests were two-sided, and *P* < 0.05 was considered statistically significant unless otherwise specified.

### 2.7 Subgroup analyses

Prespecified and data-driven subgroup analyses were performed under the random-effects model to investigate potential sources of between-dataset heterogeneity. Variables evaluated included publication period (≤ 2019 *vs* > 2019), sample size (≤ 100 *vs* > 100 animals), country (Spain, Italy, Greece, Brazil), host type (rabbit *vs* hare), serological method (IFAT, ELISA, DAT, and ICTs), antibody endpoint (IgG, total/undifferentiated antibodies, or unspecified antibody class), rabbit type (sylvatic *vs* domestic), and hare species (*L. europaeus vs L. granatensis*). The 2019 publication-year cut-off was selected solely to obtain an approximately balanced distribution of datasets between categories and was not intended to represent a biological or epidemiological threshold. Although year of sampling or study conduct would have provided a more direct measure of temporal variation, these data were incompletely or inconsistently reported in several studies; publication year was therefore used as a proxy for temporal analysis. Differences between subgroup levels were evaluated using the random-effects *Q*-between statistic and its corresponding *P*-value, with *P* < 0.05 indicating a statistically significant difference between subgroup categories. Within each subgroup, Cochran’s *Q* was used to test for residual heterogeneity, with *P* < 0.05 indicating statistically significant heterogeneity, whereas *I*^2^ quantified the proportion of total variability attributable to between-dataset heterogeneity rather than sampling error; *I*^2^ values > 75% were interpreted as indicating substantial heterogeneity. The combined rabbit-and-hare dataset was excluded from analyses requiring host-specific classification. Similarly, only rabbit datasets were used for the sylvatic-*versus*-domestic comparison, and only datasets with clearly identified hare species were included in the hare-species comparison. A continent-level subgroup analysis was not performed because the evidence originated from only four countries and was strongly geographically imbalanced, with three of the four represented countries located in Europe.

### 2.8 Analyses of host-level age and sex associations

Age and sex were analyzed separately from the prevalence subgroup analyses because these variables represented within-study host-level comparisons rather than independent study-level categories. Only studies reporting sufficient event and denominator data for both comparison groups were included. For the age analysis, animals classified in the original studies as young, juvenile, or sub-adult were harmonized into a “non-adult” category and compared with adults. For sex, males were compared with females. Random-effects pooled odds ratios (ORs) with 95% CIs were calculated for adult *versus* non-adult animals and for males *versus* females. An odds ratio of 1 represented no association with seropositivity. Between-study heterogeneity in the age- and sex-specific analyses was assessed using Cochran’s *Q* and *I*^2^; a *Q*-test *P-*value < 0.05 indicated statistically significant heterogeneity, while *I*^2^ quantified the proportion of variability attributable to between-study heterogeneity, with values > 75% interpreted as substantial. Studies reporting only the sex or age of selected seropositive animals without corresponding subgroup denominators were not included in these analyses. This restriction was necessary because several studies reported demographic descriptions without complete positive/total counts for each stratum.

### 2.9 Meta-regression

Univariable random-effects meta-regression analyses were conducted to examine whether publication year or total sample size was associated with the observed seroprevalence. Both moderators were entered as continuous variables, and analyses were performed using the method-of-moments random-effects framework on the logit event-rate scale. Regression coefficients, standard errors, 95% CIs, *Z*-statistics, and two-sided *P*-values were calculated. Residual heterogeneity was summarized using *τ*^2^, *Q*, and *I*^2^, and the *R*^2^ analogue was used to describe the proportion of between-dataset variance accounted for by each moderator. Regression coefficients were interpreted on the log-odds scale rather than as absolute percentage-point changes in seroprevalence.

### 2.10 Sensitivity analyses

Robustness of the primary pooled estimate was evaluated using leave-one-out sensitivity analysis, in which the meta-analysis was repeated after sequential removal of each dataset (Shamsi et al., 2026). The range of resulting pooled estimates was examined to determine whether the overall result was disproportionately influenced by any single dataset. An additional sensitivity analysis was conducted after excluding the single dataset with a sample size of < 10 animals, thereby restricting the analysis to datasets with at least 10 animals. Zero-event datasets were retained in the primary analysis because the absence of seropositive animals represents a valid epidemiological outcome. Their influence was already evaluated through the leave-one-out procedure, and they were therefore not excluded solely based on a zero-event count.

### 2.11 Assessment of small-study effects and publication bias

Potential small-study effects and funnel-plot asymmetry were examined using visual inspection of the funnel plot, Egger’s regression test, and Duval and Tweedie’s trim-and-fill procedure (Egger et al., 1997; Duval and Tweedie, 2000). The two-sided *P*-value from Egger’s test was used for statistical interpretation, with *P* < 0.05 considered evidence of significant funnel-plot asymmetry. Trim-and-fill analysis was conducted under the random-effects framework to estimate whether potentially missing studies would alter the pooled effect. Because funnel-plot asymmetry may arise from factors other than publication bias, particularly in the presence of substantial between-study heterogeneity, these analyses were interpreted cautiously and were not considered definitive evidence for or against publication bias.

## 3 Results

### 3.1 Study selection and characteristics of the included studies

The systematic search identified 2981 records from PubMed, Scopus, and Web of Science, with an additional 63 records identified through Google Scholar and manual reference-list screening. After removal of 1026 duplicates, 2018 records underwent title and abstract screening, of which 1993 were excluded. The full texts of 25 potentially eligible articles were assessed, and 7 articles were subsequently excluded for predefined reasons. Ultimately, 18 articles fulfilled the eligibility criteria and contributed 29 serological datasets to the quantitative synthesis (Chitimia et al., 2011; Díaz-Sáez et al., 2014; García et al., 2014; Jiménez et al., 2014; Moreno et al., 2014; Paiz et al., 2015; de la Cruz et al., 2016; Ebani et al., 2016; Ortega et al., 2017; Abbate et al., 2019; Tsakmakidis et al., 2019; Tsokana et al., 2019; Athanasiou et al., 2021; Silva et al., 2024; Barbero-Moyano et al., 2024, 2025, 2026; Cabezón et al., 2024). The study-selection process is summarized in the PRISMA 2020 flow diagram (Fig. 1).

**Fig. 1:**
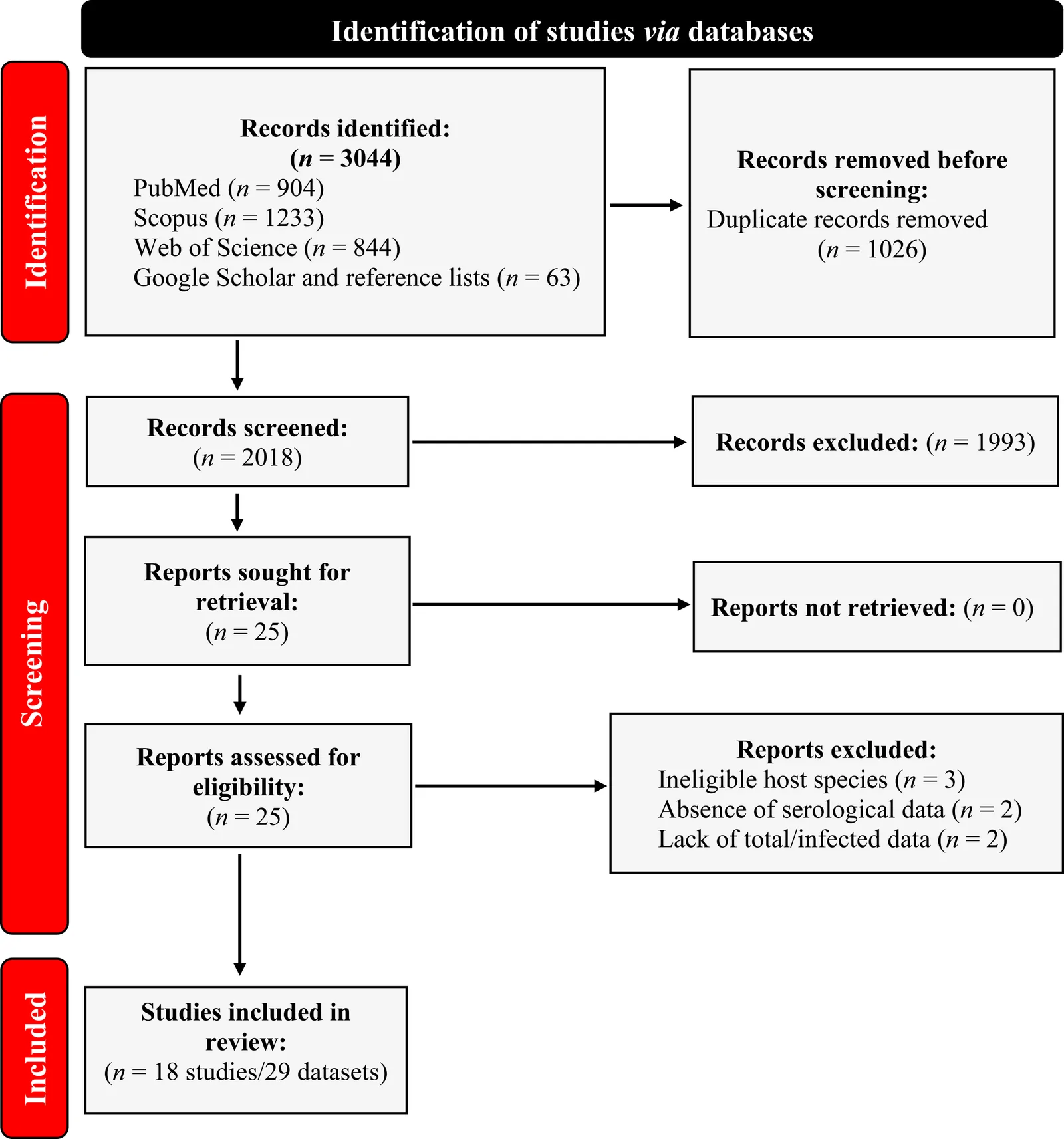
PRISMA 2020 flow diagram depicting the study-selection process for the present systematic review.

Study-level characteristics and serological definitions are presented in Table 1. Of the 29 datasets, 19 were derived from rabbits, 9 from hares, and one reported rabbits and hares as a combined population. The combined dataset was included in the overall lagomorph analysis but excluded from host-specific comparisons. The available evidence originated from only four countries (20 datasets from Spain; 5 from Greece; 2 from Italy; and 2 from Brazil), demonstrating a marked geographical concentration of the evidence. Serological testing comprised IFAT in 19 datasets, ELISA in 6, ICTs in 3, and DAT in 1. Regarding antibody endpoints, 14 datasets specifically assessed IgG, 11 reported total or undifferentiated antibodies, and four did not specify the antibody class.

**Table 1:** Characteristics of the 29 serological prevalence datasets in rabbits (*Oryctolagus cuniculus*) and hares (*Lepus* spp.) from 18 articles included in the systematic review and meta-analysis.

Dataset	Country	Sampling period	Host species	Host status	Serological method	Antibody endpoint	Positive/Total	Seroprevalence (%)^a^	Primary cut-off
Silva et al. (2024)	Brazil	NR	*O. cuniculus*	Domestic	ICT	Unspecified	6/31	19.4	NR
Paiz et al. (2015)	Brazil	2007–2013	*L. europaeus*	Sylvatic	DAT	Total/undifferentiated	1/8	12.5	≥1:320
Tsakmakidis et al. (2019) (1)	Greece	2014-2016	*O. cuniculus*	Sylvatic	ELISA	IgG	1/101	1.0	Mean negative-control OD + 3 SD
Tsakmakidis et al. (2019) (2)	Greece	May 2014 to June 2016	*O. cuniculus*	Domestic	ELISA	IgG	29/292	9.9	Mean negative-control OD + 3 SD
Athanasiou et al. (2021)	Greece	2019–2020 hunting season	*O. cuniculus*	Sylvatic	IFAT	IgG	3/72	4.2	≥1:25
Tsakmakidis et al. (2019) (3)	Greece	September 2014 to February 2016	*L. europaeus*	Sylvatic	ELISA	IgG	6/90	6.7	Mean negative-control OD + 3 SD
Tsokana et al. (2019)	Greece	Two consecutive hunting seasons (years NR)	*L. europaeus*	Sylvatic	IFAT	IgG	13/105	12.4	≥1:50
Abbate et al. (2019)	Italy	2015–2017	*O. cuniculus*	Domestic	ICT	Total/undifferentiated	1/40	2.5	Manufacturer-defined qualitative positive
Ebani et al. (2016)	Italy	2011–2015	*L. europaeus*	Sylvatic	IFAT	IgG	2/222	0.9	≥1:20
Chitimia et al. (2011)	Spain	October 2009 to March 2010	*O. cuniculus*	Sylvatic	ELISA	IgG	0/36	0	ROD >1.1
Díaz-Sáez et al. (2014)	Spain	July 2009 to October 2011	*O. cuniculus*	Sylvatic	IFAT	Total/undifferentiated	30/147	20.4	≥1:40
García et al. (2014)^b^	Spain	September 2013	*O. cuniculus*	Sylvatic	IFAT	Total/undifferentiated	52/69	75.4	≥1:25
Jiménez et al. (2014)^b^	Spain	March and October 2013	*O. cuniculus*	Sylvatic	ICT	Total/undifferentiated	11/48	22.9	Manufacturer-defined qualitative positive
Moreno et al. (2014) (1)^b^	Spain	2009–2012 outbreak survey	*O. cuniculus*	Sylvatic	IFAT	Unspecified	16/35	45.7	≥1:50
de la Cruz et al. (2016) (1)^b^	Spain	September-November 2013	*O. cuniculus*	Sylvatic	IFAT	Total/undifferentiated	107/215	49.8	≥1:50
Ortega et al. (2017) (1)^b^	Spain	September-November 2013	*O. cuniculus*	Sylvatic	IFAT	Total/undifferentiated	109/190	57.4	≥1:50
Barbero-Moyano et al. (2024) (1)	Spain	2018–2019 to 2021–2022	*O. cuniculus*	Sylvatic	IFAT	IgG	50/471	10.6	≥1:50
Cabezón et al. (2024) (1)	Spain	2014–2017 hunting seasons	*O. cuniculus*	Sylvatic	ELISA	Total/undifferentiated	31/111	27.9	≥2.8 EU
Barbero-Moyano et al. (2026) (1)	Spain	2009–2012	*O. cuniculus*	Sylvatic	IFAT	IgG	37/200	18.5	≥1:50
Barbero-Moyano et al. (2026) (2)	Spain	2012–2015	*O. cuniculus*	Sylvatic	IFAT	IgG	33/218	15.1	≥1:50
Barbero-Moyano et al. (2026) (3)	Spain	2015–2018	*O. cuniculus*	Sylvatic	IFAT	IgG	40/227	17.6	≥1:50
Barbero-Moyano et al. (2026) (4)	Spain	2018–2020	*O. cuniculus*	Sylvatic	IFAT	IgG	23/214	10.7	≥1:50
Barbero-Moyano et al. (2026) (5)	Spain	2020–2023	*O. cuniculus*	Sylvatic	IFAT	IgG	26/217	12.0	≥1:50
Cabezón et al. (2024) (2)	Spain	2012–2017 hunting seasons	*L. europaeus*	Sylvatic	ELISA	Total/undifferentiated	3/147	2.0	≥4.14 EU
Moreno et al. (2014) (2)^b^	Spain	2009–2012 outbreak survey	*L. granatensis*	Sylvatic	IFAT	Unspecified	63/85	74.1	≥1:50
de la Cruz et al. (2016) (2)^b^	Spain	September-November 2013	*L. granatensis*	Sylvatic	IFAT	Total/undifferentiated	12/69	17.4	≥1:50
Ortega et al. (2017) (2)^b^	Spain	September-November 2013	*L. granatensis*	Sylvatic	IFAT	Total/undifferentiated	13/61	21.3	≥1:50
Barbero-Moyano et al. (2024) (2)	Spain	2018–2019 to 2021–2022	*L. granatensis*	Sylvatic	IFAT	IgG	31/160	19.4	≥1:50
Barbero-Moyano et al. (2025)^c^	Spain	August 2022 to April 2023	*O. cuniculus* and *L. granatensis*	Sylvatic	IFAT	Unspecified	19/41	46.3	≥1:50

*Notes*: Numbers in parentheses in Column 1 denote distinct eligible datasets derived from the same article. The primary meta-analysis used the prespecified conservative positive threshold when multiple cut-offs or doubtful/inconclusive titres were reported.

*Abbreviations*: DAT, direct agglutination test; ELISA, enzyme-linked immunosorbent assay; EU, ELISA units; IFAT, indirect fluorescent antibody test; ICT, immunochromatographic test; NR, not reported; OD, optical density; ROD, relative optical density; SD, standard deviation.

a Seroprevalence values are based on raw positive/total counts.

b Potential overlap among some Madrid datasets remains a limitation of the evidence base.

c The combined rabbit-hare dataset from Barbero-Moyano et al. (2025) was retained only for the overall lagomorph analysis.

### 3.2 Methodological quality of the included studies

Methodological quality varied across the included studies. The most frequent limitations concerned the identification of potential confounding factors, the reporting of strategies to address confounding, and the appropriateness or completeness of statistical analyses. Overall, four articles were classified as high quality and 14 as moderate quality (Supplementary Table S1).

### 3.3 Overall pooled seroprevalence of anti-*Leishmania* spp. antibodies in lagomorphs

Based on the available published evidence, the random-effects pooled seroprevalence of anti-*Leishmania* spp. antibodies in lagomorphs was 17.3% (95% CI: 12.0–24.2%) across the 29 datasets (Fig. 2). Considerable between-dataset heterogeneity was observed (*Q* = 563.424, *df* = 28, *P* < 0.001; *I*^2^ =95.0%; *τ*^2^ = 1.171; *τ* = 1.082), supporting the use of a random-effects model and indicating substantial variability among the available populations (Table 2).

**Fig. 2:**
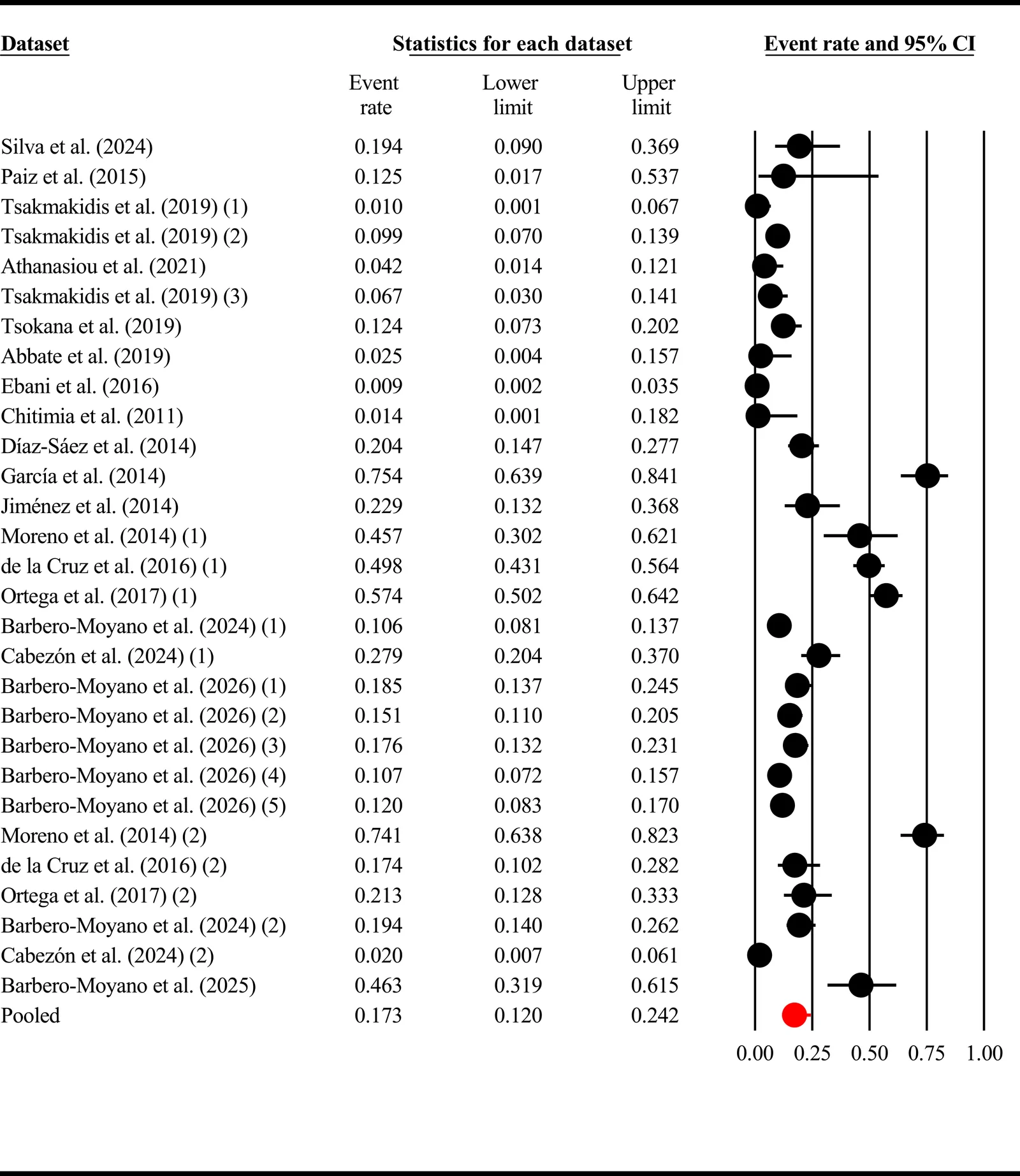
Forest plot showing the pooled seroprevalence of anti-*Leishmania* spp. antibodies in lagomorphs. Each horizontal black line represents the 95% confidence interval (CI) for the seroprevalence reported in an individual dataset. The black circles indicate the point estimate (event rate) for each dataset. The red circle summarizes the pooled seroprevalence and its 95% CI. For the zero-event dataset, the plotted event rate reflects the 0.5 continuity correction used for logit-based meta-analysis; the raw seroprevalence data are provided in Table 1. A number in parentheses after a reference indicates a distinct eligible dataset derived from the same article; details of these datasets are provided in Table 1.

**Table 2:** Random-effects subgroup analyses of pooled anti-*Leishmania* spp. seroprevalence based on the available published evidence.

Variable	Subgroup	No. of datasets	Pooled seroprevalence, % (95% CI)	*Q* (*df*)	*P* for Q	*I*^2^ (%)	*Q*-between (*df*)	*P*-between
Overall	All lagomorph datasets	29	17.3 (12.0–24.2)	563.424 (28)	<0.001	95.0	–	–
Publication year	≤ 2019	17	19.0 (10.9–31.0)	349.449 (16)	<0.001	95.4	0.484 (1)	0.487
	> 2019	12	15.3 (11.3–20.3)	75.154 (11)	<0.001	85.4		
Sample size	≤ 100	13	21.9 (11.2–38.6)	165.445 (12)	<0.001	92.7	1.225 (1)	0.268
	> 100	16	14.1 (9.1–21.4)	354.259 (15)	<0.001	95.8		
Country	Brazil	2	18.1 (8.9–33.5)	0.199 (1)	0.655	0	33.995 (3)	<0.001
	Greece	5	7.6 (4.6–12.2)	9.489 (4)	0.050	57.8		
	Italy	2	1.3 (0.4–3.8)	0.703 (1)	0.402	0		
	Spain	20	24.6 (16.9–34.5)	448.863 (19)	<0.001	95.8		
Host type	Hare	9	12.4 (5.0–27.4)	148.844 (8)	<0.001	94.6	0.722 (1)	0.396
	Rabbit	19	18.6 (12.1–27.3)	403.155 (18)	<0.001	95.5		
Serological method	IFAT	19	22.9 (15.2–33.0)	469.240 (18)	<0.001	96.2	7.654 (3)	0.054
	ELISA	6	6.0 (2.3–14.5)	47.213 (5)	<0.001	89.4		
	ICT	3	15.3 (6.4–32.4)	5.256 (2)	0.072	61.9		
	DAT	1	12.5 (1.7–53.7)	NA	NA	NA		
Antibody endpoint	IgG	14	10.9 (8.4–14.0)	55.010 (13)	<0.001	76.4	20.353 (2)	<0.001
	Total/undifferentiated antibodies	11	25.0 (15.0–38.6)	164.128 (10)	<0.001	93.9		
	Antibody class unspecified	4	46.8 (25.1–69.8)	27.062 (3)	<0.001	88.9		
Rabbit type	Domestic	3	10.7 (5.0–21.2)	4.830 (2)	0.089	58.6	2.414 (1)	0.120
	Sylvatic	16	20.5 (13.1–30.8)	364.656 (15)	<0.001	95.9		
Hare species	*Lepus europaeus*	5	4.7 (1.8–12.0)	18.586 (4)	0.001	78.5	6.995 (1)	0.008
	*Lepus granatensis*	4	30.9 (11.1–61.6)	73.985 (3)	<0.001	95.9		

*Note*: Estimates are from random-effects models using logit-transformed event rates. *Q*, *df*, *P* for *Q*, and *I*^2^ describe within-subgroup heterogeneity; *Q*-between and *P*-between are from the random-effects test of subgroup differences and are shown on the first row of each variable.

*Abbreviations*: *df*, degrees of freedom; CI, confidence interval; DAT, direct agglutination test; ELISA, enzyme-linked immunosorbent assay; IFAT, indirect fluorescent antibody test; ICT, immunochromatographic test; NA, heterogeneity could not be meaningfully estimated from a single dataset.

### 3.4 Subgroup analyses by publication period and sample size

When datasets were categorized according to publication year, the pooled seroprevalence was 19.0% (95% CI: 10.9–31.0%) among 17 datasets published in or before 2019 and 15.3% (95% CI: 11.3–20.3%) among 12 datasets published after 2019 (Supplementary Fig. S1). The difference between the two periods was not statistically significant (*Q*-between = 0.484, *df* = 1, *P* = 0.487). Substantial residual heterogeneity remained in both groups (*I*^2^ = 95.4% and 85.4%, respectively) (Table 2).

Similarly, datasets with ≤ 100 animals showed a numerically higher pooled seroprevalence of 21.9% (95% CI: 11.2–38.6%) compared with 14.1% (95% CI: 9.1–21.4%) among datasets with > 100 animals (Supplementary Fig. S2). However, this difference was not statistically significant (*Q*-between = 1.225, *df* = 1, *P* = 0.268), and heterogeneity remained high in both categories (*I*^2^ = 92.7% and 95.8%, respectively) (Table 2).

### 3.5 Subgroup analysis by country

The geographical distribution of the available evidence and country-specific pooled estimates are illustrated in Fig. 3. Pooled seroprevalence differed significantly among the four represented countries (*Q*-between = 33.995, *df* = 3, *P* < 0.001). Spain had the highest pooled estimate at 24.6% (95% CI: 16.9–34.5%; 20 datasets), followed by Brazil at 18.1% (95% CI: 8.9–33.5%; 2 datasets), Greece at 7.6% (95% CI: 4.6–12.2%; 5 datasets), and Italy at 1.3% (95% CI: 0.4–3.8%; 2 datasets) (Supplementary Fig. S3). Marked heterogeneity persisted among the Spanish datasets (*I*^2^ = 95.8%), whereas moderate heterogeneity was observed in Greece (*I*^2^ = 57.8%). Estimates of heterogeneity for Brazil and Italy were based on only two datasets each and should therefore be interpreted cautiously (Table 2).

**Fig. 3:**
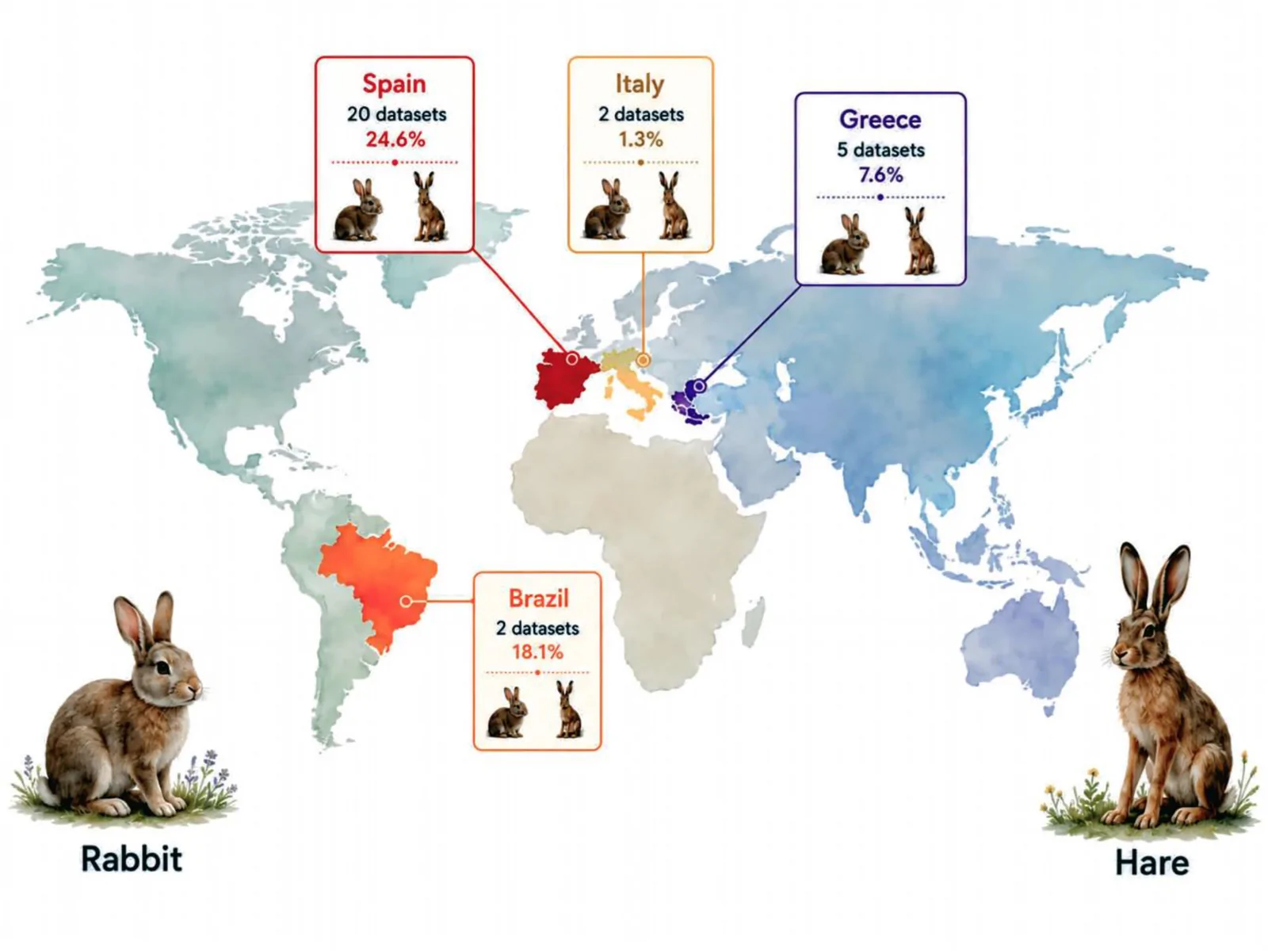
Geographical distribution of available published serological evidence of *Leishmania* spp. exposure in rabbits and hares. The map shows the four represented countries, the number of eligible datasets, and country-specific random-effects pooled seroprevalence. The figure is schematic; highlighted countries are slightly enlarged for visibility and are not strictly proportional in size.

### 3.6 Subgroup analysis by host type

The combined rabbit-hare dataset was excluded from the host-specific analysis, leaving 28 datasets. The pooled seroprevalence was 18.6% (95% CI: 12.1–27.3%) across 19 rabbit datasets and 12.4% (95% CI: 5.0–27.4%) across nine hare datasets (Supplementary Fig. S4). The between-host difference was not statistically significant (*Q*-between = 0.722, *df* = 1, *P* = 0.396). Considerable heterogeneity persisted in both rabbits (*I*^2^ = 95.5%) and hares (*I*^2^ = 94.6%) (Table 2).

### 3.7 Subgroup analysis by serological method

Seroprevalence varied numerically according to the serological assay. IFAT yielded the highest pooled estimate at 22.9% (95% CI: 15.2–33.0%; 19 datasets), followed by ICTs at 15.3% (95% CI: 6.4–32.4%; 3 datasets), DAT at 12.5% (95% CI: 1.7–53.7%; 1 dataset), and ELISA at 6.0% (95% CI: 2.3–14.5%; 6 datasets) (Supplementary Fig. S5). The overall between-method difference approached but did not reach statistical significance under the random-effects model (*Q*-between = 7.654, *df* = 3, *P* = 0.054). Considerable heterogeneity remained within the IFAT (*I*^2^ = 96.2%) and ELISA (*I*^2^ = 89.4%) groups, whereas moderate heterogeneity was observed among the ICT datasets (*I*^2^ = 61.9%); the DAT estimate was derived from a single dataset (Table 2).

### 3.8 Subgroup analysis by antibody endpoint

A significant difference was observed according to the antibody endpoint evaluated (*Q*-between = 20.353, *df* = 2, *P* < 0.001). Studies specifically assessing IgG had the lowest pooled seroprevalence at 10.9% (95% CI: 8.4–14.0%; 14 datasets). Studies reporting total or undifferentiated antibodies yielded a pooled estimate of 25.0% (95% CI: 15.0–38.6%; 11 datasets), whereas studies in which the antibody class was unspecified showed the highest estimate at 46.8% (95% CI: 25.1–69.8%; 4 datasets) (Supplementary Fig. S6). Residual heterogeneity remained substantial within all three categories (*I*^2^ = 76.4%, 93.9%, and 88.9%, respectively) (Table 2).

### 3.9 Subgroup analyses by rabbit type and hare species

Among rabbit datasets, sylvatic rabbits showed a higher numerical pooled seroprevalence of 20.5% (95% CI: 13.1–30.8%; 16 datasets) compared with 10.7% (95% CI: 5.0–21.2%; 3 datasets) in domestic rabbits (Supplementary Fig. S7). However, the difference was not statistically significant (*Q*-between = 2.414, *df* = 1, *P* = 0.120). Heterogeneity was substantial among sylvatic rabbit datasets (*I*^2^ = 95.9%) and moderate among domestic rabbit datasets (*I*^2^ = 58.6%) (Table 2).

In contrast, seroprevalence differed significantly between the two hare species (*Q*-between = 6.995, *df* = 1, *P* = 0.008). The pooled estimate for *L. europaeus* was 4.7% (95% CI: 1.8–12.0%; 5 datasets), compared with 30.9% (95% CI: 11.1–61.6%; 4 datasets) for *L. granatensis* (Supplementary Fig. S8). Considerable residual heterogeneity was observed in both species (*L. europaeus*: *I*^2^ = 78.5%; *L. granatensis*: *I*^2^ = 95.9%) (Table 2).

### 3.10 Host-level associations with age and sex

The results of the host-level analyses of age and sex are summarized in Table 3. Age and sex were evaluated separately using random-effects OR meta-analyses because these variables represented within-study host-level comparisons rather than conventional prevalence subgroups. Four studies provided analysable age-stratified data. Adults showed numerically higher odds of anti-*Leishmania* spp. seropositivity than non-adult animals, although the association did not reach statistical significance (OR = 1.69, 95% CI: 0.92–3.10, *P* = 0.089) (Supplementary Fig. S9). Between-study heterogeneity was low to moderate (*Q* = 4.474, *df* = 3, *P* = 0.215; *I*^2^ = 32.9%). Five studies contributed sex-stratified data. No association was observed between sex and seropositivity (male *vs* female: OR = 1.00, 95% CI: 0.68–1.47, *P* = 1.000) (Supplementary Fig. S10), with no detectable between-study heterogeneity (*Q* = 3.518, *df* = 4, *P* = 0.475; *I*^2^ = 0%).

**Table 3:** Random-effects meta-analysis of host-level factors associated with anti-*Leishmania* spp. seropositivity.

Host-level factor	Comparison	No. of studies	OR (95% CI)	*Z*	*P*	*Q* (*df*)	*P* for *Q*	*I*^2^ (%)	*τ*^2^
Age	Adult *vs* non-adult	4	1.69 (0.92–3.10)	1.700	0.089	4.474 (3)	0.215	32.9	0.124
Sex	Male *vs* female	5	1.00 (0.68–1.47)	0.000	1.000	3.518 (4)	0.475	0.0	0.000

*Notes*: Odds ratios are from random-effects models. OR >1 indicates higher odds of anti-*Leishmania* spp. seropositivity in the first group named in each comparison. For age, the non-adult category combined young, juvenile, and subadult strata as reported in the contributing studies. *Q*, *df*, *P* for *Q*, *I*^2^, and *τ*^2^ describe between-study heterogeneity. These host-level analyses are based on the limited studies reporting exact stratum-specific positive and total counts and should be interpreted as exploratory.

*Abbreviations*: *df*, degrees of freedom; CI, confidence interval; OR, odds ratio.

### 3.11 Sensitivity analyses

The leave-one-out analysis demonstrated that the primary pooled estimate was robust to sequential exclusion of individual datasets. The recalculated pooled seroprevalence ranged from 16.0% (95% CI: 11.2–22.3%) to 18.7% (95% CI: 13.1–25.9%), compared with the primary estimate of 17.3% (95% CI: 12.0–24.2%), indicating that no single dataset materially influenced the overall result (Supplementary Fig. S11). A second sensitivity analysis excluded the only dataset with a sample size of < 10 animals. The pooled seroprevalence across the remaining 28 datasets was 17.4% (95% CI: 12.0–24.4%), essentially unchanged from the primary analysis (Supplementary Fig. S12). Considerable heterogeneity persisted (*Q* = 562.865, *df* = 27, *P* < 0.001; *I*^2^ = 95.2%; *τ*^2^ = 1.175), confirming that the small dataset was not responsible for either the pooled estimate or the high heterogeneity.

### 3.12 Meta-regression

Univariable random-effects meta-regression did not identify a statistically significant association between publication year and seroprevalence (Fig. 4A), although a borderline negative trend was observed (coefficient = −0.0729, SE = 0.0398, 95% CI: −0.1509 to 0.0052, *Z* = −1.83, *P* = 0.067). Publication year accounted for approximately 29% of the between-dataset variance, but substantial residual heterogeneity remained (*τ*^2^ = 0.830; *I*^2^ = 93.0%). Sample size was also not significantly associated with seroprevalence (coefficient = −0.0022, SE = 0.0021, 95% CI: −0.0063 to 0.0019, *Z* = −1.07, *P* = 0.286) (Fig. 4B). Sample size explained approximately 8% of the between-dataset variance, with substantial unexplained heterogeneity persisting (*τ*^2^ = 1.077; *I*^2^ = 94.4%).

**Fig. 4:**
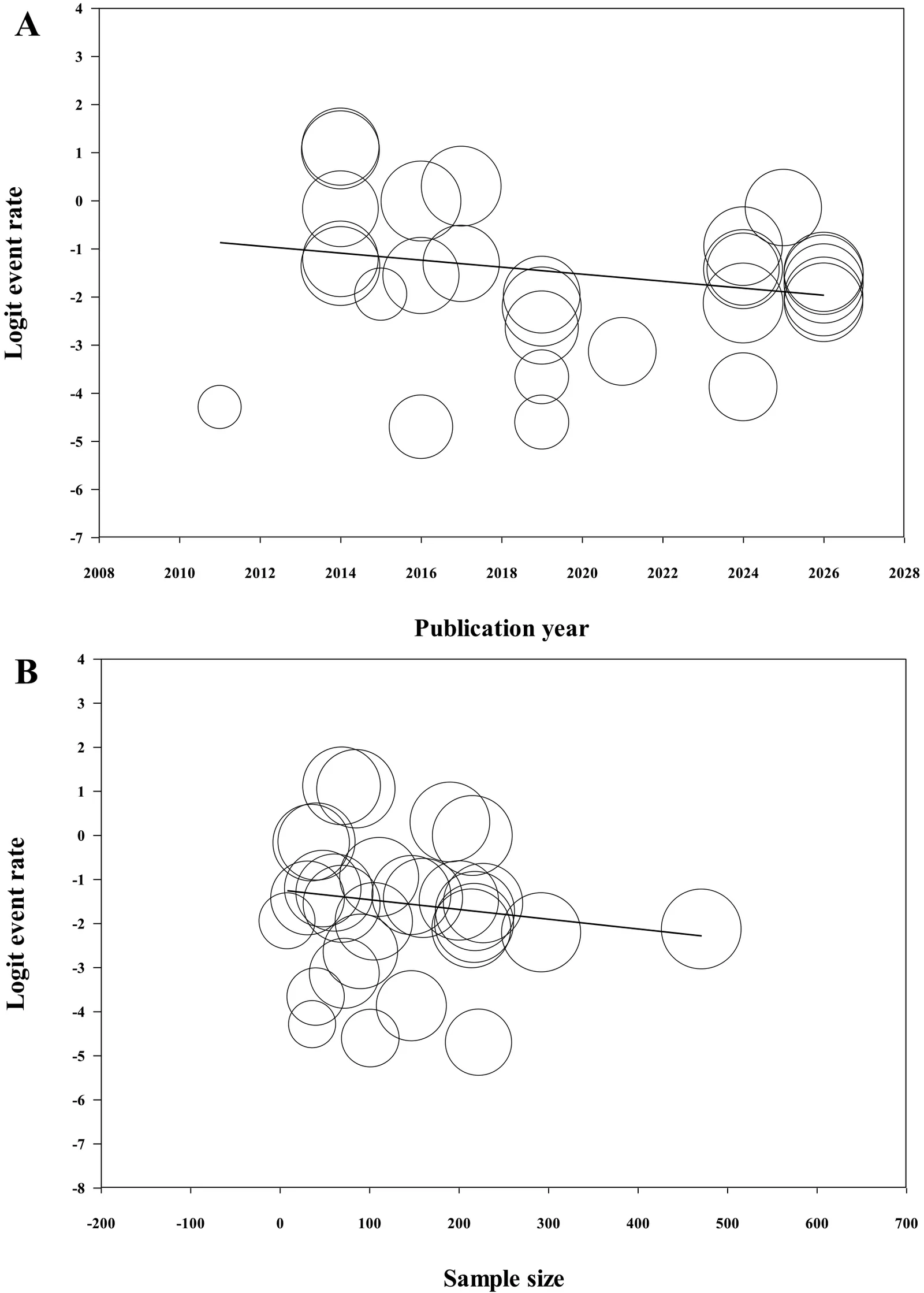
Bubble plots of univariable random-effects meta-regression examining the association between the logit-transformed seroprevalence of anti-*Leishmania* spp. antibodies in lagomorphs and publication year (**A**) and sample size (**B**). For publication year, the regression coefficient was −0.0729 (SE = 0.0398; 95% CI: −0.1509 to 0.0052; *Z* = −1.83; *P* = 0.067), with *R*^2^ ≈ 29%, residual *τ*^2^ = 0.830, and residual *I*^2^ = 93.0%. For sample size, the regression coefficient was −0.0022 (SE = 0.0021; 95% CI: −0.0063 to 0.0019; *Z* = −1.07; *P* = 0.286), with *R*^2^ ≈ 8%, residual *τ*^2^ = 1.077, and residual *I*^2^ = 94.4%. Regression coefficients are expressed on the logit event-rate scale. Each bubble represents one dataset, with bubble size reflecting its relative meta-analytic weight; the solid line represents the fitted meta-regression slope.

### 3.13 Small-study effects and publication bias

Egger’s regression test showed no statistically significant funnel-plot asymmetry using the prespecified two-sided criterion (intercept = −3.1108, SE = 1.7681, 95% CI: −6.7387 to 0.5171, *t* = −1.759, *df* = 27, *P* = 0.090). Consistently, Duval and Tweedie’s trim-and-fill procedure imputed no missing studies, and the adjusted random-effects estimate remained identical to the observed pooled estimate (17.3%, 95% CI: 12.0–24.2%). These findings did not indicate a material effect of publication bias on the pooled estimate, although interpretation remains cautious given the substantial heterogeneity across datasets (Supplementary Fig. S13).

## 4 Discussion

This systematic review and meta-analysis provides a quantitative synthesis of the available published serological evidence of *Leishmania* spp. exposure in rabbits and hares. The pooled seroprevalence was 17.3%, but the very high heterogeneity (*I*^2^ = 95.0%) indicates that this value should be interpreted as an overall summary of heterogeneous published populations rather than a uniform estimate applicable across geographical settings. The magnitude of serological exposure observed in lagomorphs is epidemiologically relevant; notably, a recent meta-analysis in dogs estimated an overall *L. infantum* prevalence of approximately 15%, although direct numerical comparison is inappropriate because host populations, diagnostic methods, geographical coverage, and definitions of positivity differ substantially (Priolo et al., 2024). The robustness of the present estimate was supported by both leave-one-out analysis and exclusion of the dataset with fewer than 10 animals.

Country was one of the strongest sources of variation, with pooled estimates ranging from 1.3% in Italy to 24.6% in Spain. This pattern is consistent with the pronounced geographical heterogeneity previously reported for *Leishmania* spp. in sylvatic hosts and likely reflects differences in local vector abundance, host communities, environmental conditions, and transmission intensity (Azami-Conesa et al., 2021). Urban and peri-urban planning may also influence local transmission dynamics by modifying land use and creating ecological conditions that affect vector and reservoir-host populations. In the southwestern Madrid outbreak in Spain, the development of the BosqueSur peri-urban green area and other nearby land-use changes were considered potentially relevant to the altered local ecology of leishmaniasis transmission (Gómez-Barroso et al., 2015). Recent surveillance in southern Spain found anti-*Leishmania* spp. antibodies in both sylvatic rabbits and Iberian hares and demonstrated marked spatial clustering of exposure, while long-term monitoring from 2009 to 2023 further confirmed persistent but spatially heterogeneous circulation among Spanish sylvatic rabbits (Barbero-Moyano et al., 2024, 2026). In contrast, a Greek survey reported relatively low seropositivity in rabbits and hares, and a study of *L. europaeus* in central Italy detected anti-*Leishmania* spp. antibodies in only 0.9% of examined hares (Ebani et al., 2016; Tsakmakidis et al., 2019). The Brazilian evidence remains limited, although a recent study detected anti-*Leishmania* spp. antibodies in 19.35% of domestic rabbits (Silva et al., 2024). These observations broadly support the country effect detected here, but the strong predominance of Spanish datasets prevents interpretation of these estimates as representative national prevalences.

Overall seroprevalence did not differ significantly between rabbits and hares, and the higher estimate observed in sylvatic compared with domestic rabbits was also not statistically significant. However, a significant difference emerged between hare species, with *L. granatensis* showing substantially higher pooled seroprevalence than *L. europaeus*. This finding is biologically interesting because *L. granatensis* has been implicated in *L. infantum* transmission in Spain, including direct xenodiagnostic evidence demonstrating transmission from naturally infected hares to *Phlebotomus perniciosus* (Molina et al., 2012). Recent Spanish studies have also reinforced the epidemiological importance of both Iberian hares and sylvatic rabbits within local transmission systems (Barbero-Moyano et al., 2024; Martín-Sánchez et al., 2024). Nevertheless, the species effect in the present meta-analysis should not be interpreted as evidence of intrinsically greater susceptibility of *L. granatensis*, because its datasets were concentrated in Spain whereas *L. europaeus* was represented largely by other geographical settings. Species and geography are therefore difficult to disentangle with the available evidence.

Methodological variation also contributed importantly to the observed differences. Seroprevalence varied from 6.0% with ELISA to 22.9% with IFAT, although the between-method comparison narrowly missed statistical significance. In contrast, antibody endpoint was significantly associated with the pooled estimate, with lower seroprevalence among studies specifically measuring IgG than among studies reporting total/undifferentiated or unspecified antibody classes. Differences in antigen preparation, detection systems, titre thresholds, and interpretation criteria can materially influence serological classification. In lagomorphs, changing the IFAT threshold from a more sensitive to a more conservative cut-off has been shown to alter diagnostic interpretation, while recent inter-laboratory work in veterinary *L. infantum* serology demonstrated meaningful variation between IFAT and ELISA implementation and performance (de la Cruz et al., 2016; Mahachi et al., 2024). Thus, the significant antibody-endpoint effect should primarily be interpreted as evidence of methodological heterogeneity rather than a biological difference in exposure.

The distinction between serological exposure and active infection is particularly important. In a recent large Spanish lagomorph study, seroprevalence was 12.8%, whereas molecular detection reached 59.0%, with only slight agreement between IFAT and qPCR and no correlation between antibody titre and estimated parasite burden (Barbero-Moyano et al., 2024). This discrepancy may partly reflect the high analytical sensitivity of the specific qPCR assay used for direct detection of low amounts of *L. infantum* DNA in lagomorph tissues; previous work in sylvatic leporids showed that qPCR detected a higher proportion of positive animals than IFAT and had a very low analytical detection limit (Ortega et al., 2017). Nevertheless, molecular and serological methods detect different biological targets, and their diagnostic performance may vary according to the assay, sampled tissue, parasite distribution, and stage of infection. Therefore, detection of antibodies cannot by itself establish current parasite carriage or reservoir competence. Evidence for a reservoir role requires complementary ecological, molecular, and preferably vector-infectivity data, as demonstrated by xenodiagnostic investigations in Iberian hares and subsequent work in sylvatic rabbits (Molina et al., 2012; Martín-Sánchez et al., 2024; Tsakmakidis et al., 2024).

Neither publication year nor sample size was significantly associated with seroprevalence in meta-regression, although publication year showed a borderline decreasing trend. These findings should not be interpreted as evidence of stable temporal transmission because publication year is only an indirect proxy for the actual sampling period. Long-term surveillance in southern Spain has demonstrated persistence of *L. infantum* exposure together with substantial spatial and temporal variability in sylvatic rabbits (Barbero-Moyano et al., 2026). Likewise, the absence of significant pooled associations with age or sex suggests that these variables were not consistent predictors across the available datasets, rather than proving that they have no influence within particular populations. For example, recent Spanish surveillance found no significant sex-associated difference but identified several ecological and environmental correlates of exposure (Barbero-Moyano et al., 2024).

The principal strengths of this study include the use of a predefined conservative seropositivity rule, random-effects modelling, multiple subgroup analyses, meta-regression, host-level comparative analyses, sensitivity analyses, and complementary assessment of small-study effects. Nevertheless, several limitations are important. First, the evidence was geographically restricted to only four countries, with 20 of 29 datasets originating from Spain. Secondly, some Spanish datasets, particularly those from the Madrid focus, may involve partially overlapping animal populations, and complete independence could not always be verified. Thirdly, several subgroup categories contained few datasets, reducing precision and increasing the possibility of confounding between country, host species, assay, and antibody endpoint. Fourthly, serological methods and cut-offs were not standardized across studies, an established problem in veterinary *Leishmania* spp. serology (de la Cruz et al., 2016; Mahachi et al., 2024). Fifthly, sampling year was inconsistently reported, necessitating use of publication year for temporal analyses. Finally, the review protocol was not prospectively registered. These limitations reinforce the need for standardized, geographically broader investigations using harmonized serological definitions and complementary molecular and vector-based evidence (Azami-Conesa et al., 2021; Tsakmakidis et al., 2024).

## 5 Conclusions

The available published evidence indicates substantial but highly heterogeneous serological exposure to *Leishmania* spp. among rabbits and hares. Country, antibody endpoint, and hare species were associated with significant variation, whereas host type, rabbit husbandry status, publication period, sample size, and host age and sex were not consistent predictors. These findings support the epidemiological relevance of lagomorph exposure within *Leishmania* spp. transmission systems, particularly in Mediterranean settings, but seropositivity alone should not be interpreted as evidence of active infection or reservoir competence. Because the current evidence is geographically concentrated and methodologically heterogeneous, the pooled estimate should not be regarded as a worldwide seroprevalence. Future studies should prioritize standardized serological protocols, broader geographical coverage, clearly reported sampling periods, and integration of serological, molecular, ecological, and vector-infectivity data.

## Ethical approval

Not applicable.

## CRediT authorship contribution statement

**Ali Asghari:** Conceptualization, Methodology, Investigation, Formal analysis, Writing - original draft, Writing - review & editing. **Giovanni Sgroi:** Investigation, Data curation, Writing - review & editing. **Zaid Hassan Mohsin Alsafi:** Investigation, Data curation, Writing - review & editing. **Laya Shamsi:** Investigation, Data curation, Writing - review & editing.

## Data availability

All data generated or analyzed during this study are included in this published article and its supplementary files. The raw data extracted from the publications included in this systematic review and meta-analysis and used in the quantitative analyses are provided in Supplementary Table S2.

## Statement on the use of AI-assisted technologies

During the preparation of this manuscript, the authors used ChatGPT (OpenAI) solely to support language editing, paraphrasing, stylistic polishing, and visual refinement of selected figures. Generative artificial intelligence (AI) was not used to generate, modify, fabricate, analyse, or interpret study data, nor was it used to make decisions regarding study eligibility, quality assessment, statistical analyses, or scientific conclusions. All scientific information and numerical values presented in the manuscript and figures were derived from the original studies and the authors’ analyses. Any AI-assisted output was critically reviewed, verified against the original sources and study results, and revised by the authors before inclusion in the manuscript. The authors take full responsibility for the content of the article.

## Funding

This research received no specific grant from any funding agency in the public, commercial, or not-for-profit sectors.

## Declaration of competing interests

The authors declare that they have no known competing financial interests or personal relationships that could have appeared to influence the work reported in this paper.
