# Supplementary material for: Serological evidence of Leishmania spp. exposure in rabbits and hares: A systematic review and meta-analysis
Source: Curr Res Parasitol Vector Borne Dis. 2026 Oct 27;10:100453. doi: 10.1016/j.crpvbd.2026.100453 (PMC13744300; doi:10.1016/j.crpvbd.2026.100453)
Supplement: Supplementary file 1 [file mmc1.pdf]

Supplementary file 1

Supplementary Table S1. Joanna Briggs Institute (JBI) critical appraisal checklist applied for included studies.

Cross-sectional									
**Reference**	**Q1**	**Q2**	**Q3**	**Q4**	**Q5**	**Q6**	**Q7**	**Q8**	**Overall quality**
de la Cruz et al. (2016)	Yes	Yes	Yes	Yes	No	No	Yes	No	5
Tsokana et al. (2019)	Yes	Yes	Yes	Yes	No	No	Yes	No	5
Tsakmakidis et al. (2019)	Yes	Yes	Yes	Yes	No	No	Yes	No	5
Barbero-Moyano et al. (2026)	Yes	Yes	Yes	Yes	Unclear	No	Yes	Yes	6.5
Ebani et al. (2016)	Yes	Yes	Yes	Yes	No	No	Yes	No	5
Díaz-Sáez et al. (2014)	Yes	Yes	Yes	Yes	Unclear	No	Yes	No	5.5
García et al. (2014)	Yes	Yes	Yes	Yes	Unclear	No	Yes	No	5.5
Cabezón et al. (2024)	Yes	Yes	Yes	Yes	No	No	Yes	Yes	6
Athanasiou et al. (2021)	Yes	Yes	Yes	Yes	No	No	Yes	Yes	6
Silva et al. (2024)	Yes	Yes	Yes	Yes	Unclear	No	Yes	Yes	6.5
Ortega et al. (2017)	Yes	Yes	Yes	Yes	No	No	Yes	No	5
Paiz et al. (2015)	Yes	Yes	Yes	Yes	No	No	Yes	Yes	6
Jiménez et al. (2014)	Yes	Yes	Yes	Yes	Unclear	No	Yes	Yes	6.5
Abbate et al. (2019)	Yes	Yes	Yes	Yes	No	No	Yes	No	5
Barbero-Moyano et al. (2024)	Yes	Yes	Yes	Yes	Unclear	No	Yes	Yes	6.5
Chitimia et al. (2011)	Yes	Yes	Yes	Yes	Unclear	No	Yes	No	5.5
Moreno et al. (2014)	Yes	Yes	Yes	Yes	No	No	Yes	Yes	6
Barbero-Moyano et al. (2025)	Yes	Yes	Yes	Yes	No	No	Yes	Yes	6

Cross-sectional questions:

Q1. Were the criteria for inclusion in the sample clearly defined?

Q2. Were the study subjects and the setting described in detail?

Q3. Was exposure measured in a valid and reliable way?

Q4. Were objective, standard criteria used for measurement of the condition?

Q5. Were confounding factors identified?

Q6. Were strategies to deal with confounding factors stated?

Q7. Were the outcomes measured in a valid and reliable way?

Q8. Was appropriate statistical analysis used?

Supplementary Table S2. Raw data extracted from the publications included in the systematic review and meta-analysis.

Table S2A presents the dataset-level serological counts and assay definitions used in the prevalence meta-analysis. Table S2B presents the exact stratum-specific event and denominator data used in the host-level age and sex meta-analyses.

Supplementary Table S2A. Dataset-level raw serological data and assay definitions used to estimate anti-*Leishmania* spp. seroprevalence in rabbits (*Oryctolagus cuniculus)* and hares (*Lepus* spp.).

**Study and host characteristics**					**Serological assessment**			**Raw prevalence outcome**		
**Dataset**	**Sampling period**	**Country**	**Host species**	**Host status**	**Serological method**	**Antibody endpoint**	**Primary cut-off / positivity criterion**	**Seropositive (*n*)**	**Total examined (*n*)**	**Seroprevalence (%)**
Chitimia et al. (2011)	October 2009-March 2010	Spain	*O. cuniculus*	Sylvatic	Commercial ELISA (Civtest Canis Leishmania®, Hipra)	IgG	ROD >1.1 positive; 0.9–1.1 inconclusive; <0.9 negative. One reported inconclusive result was excluded from the positive count.	0	36	0.0
Díaz-Sáez et al. (2014)	July 2009-October 2011	Spain	*O. cuniculus*	Sylvatic	IFAT	Total/ undifferentiated	≥1:40 (conservative; 42/147 had titres ≥1:20; no standardized rabbit cut-off available)	30	147	20.4
García et al. (2014)	September 2013	Spain	*O. cuniculus*	Sylvatic	IFAT	Total/ undifferentiated	≥1:25	52	69	75.4
Jiménez et al. (2014)	March and October 2013	Spain	*O. cuniculus*	Sylvatic	rK39 ICT (Kalazar Detect® Canine Rapid Test, InBios)	Total/ undifferentiated	Manufacturer-defined qualitative positive result; no numeric cut-off reported	11	48	22.9
Moreno et al. (2014) (1)	2009-2012 outbreak survey	Spain	*O. cuniculus*	Sylvatic	IFAT	Unspecified	Positive titre ≥1:50; sera reactive only at 1:25 were excluded from the positive count	16	35	45.7
Moreno et al. (2014) (2)	2009-2012 outbreak survey	Spain	*L. granatensis*	Sylvatic	IFAT	Unspecified	Positive titre ≥1:50; sera reactive only at 1:25 were excluded from the positive count	63	85	74.1
Paiz et al. (2015)	2007-2013	Brazil	*L. europaeus*	Sylvatic	DAT	Total/ undifferentiated	≥1:320 (conservative predefined cut-off)	1	8	12.5
de la Cruz et al. (2016) (1)	September-November 2013	Spain	*O. cuniculus*	Sylvatic	IFAT	Total/ undifferentiated	≥1:50 (conservative; 35 inconclusive titres at 1:25 excluded)	107	215	49.8
de la Cruz et al. (2016) (2)	September-November 2013	Spain	*L. granatensis*	Sylvatic	IFAT	Total/ undifferentiated	≥1:50 (conservative; 9 inconclusive titres at 1:25 excluded)	12	69	17.4

Study and host characteristics					Serological assessment			Raw prevalence outcome		
**Dataset**	**Sampling period**	**Country**	**Host species**	**Host status**	**Serological method**	**Antibody endpoint**	**Primary cut-off / positivity criterion**	**Seropositive (*n*)**	**Total examined (*n*)**	**Seroprevalence (%)**
Ebani et al. (2016)	2011-2015	Italy	*L. europaeus*	Sylvatic	IFAT	IgG	≥1:20	2	222	0.9
Ortega et al. (2017) (1)	September-November 2013	Spain	*O. cuniculus*	Sylvatic	IFAT	Total/ undifferentiated	≥1:50; initially screened at 1:25	109	190	57.4
Ortega et al. (2017) (2)	September-November 2013	Spain	*L. granatensis*	Sylvatic	IFAT	Total/ undifferentiated	≥1:50; initially screened at 1:25	13	61	21.3
Tsokana et al. (2019)	Two consecutive hunting seasons (years not reported)	Greece	*L. europaeus*	Sylvatic	IFAT	IgG	≥1:50 (lowest reported positive titre; explicit cut-off not stated)	13	105	12.4
Tsakmakidis et al. (2019) (1)	2014-2016; two hunting seasons (August-March)	Greece	*O. cuniculus*	Sylvatic	In-house ELISA	IgG	Mean OD of negative controls + 3 SD	1	101	1.0
Tsakmakidis et al. (2019) (2)	11 May 2014-6 June 2016	Greece	*O. cuniculus*	Domestic	In-house ELISA	IgG	Mean OD of negative controls + 3 SD	29	292	9.9
Tsakmakidis et al. (2019) (3)	24 September 2014-22 February 2016; three hunting seasons	Greece	*L. europaeus*	Sylvatic	In-house ELISA	IgG	Mean OD of negative controls + 3 SD	6	90	6.7
Abbate et al. (2019)	2015-2017	Italy	*O. cuniculus*	Domestic	rK39 ICT (Leishmania Dipstick rapid test®, APACOR)	Total/ undifferentiated	Manufacturer-defined qualitative positive result; no numeric cut-off reported	1	40	2.5
Athanasiou et al. (2021)	Hunting season 2019-2020	Greece	*O. cuniculus*	Sylvatic	IFAT	IgG	≥1:25	3	72	4.2
Cabezón et al. (2024) (1)	October-February, 2014-2017	Spain	*O. cuniculus*	Sylvatic	In-house indirect ELISA	Total/ undifferentiated	Positive ≥2.8 EU; doubtful 2.0-<2.8 EU excluded	31	111	27.9
Cabezón et al. (2024) (2)	October-March, 2012-2017	Spain	*L. europaeus*	Sylvatic	In-house indirect ELISA	Total/ undifferentiated	Positive ≥4.14 EU; doubtful 2.43-<4.14 EU excluded	3	147	2.0
Silva et al. (2024)	Not reported	Brazil	*O. cuniculus*	Domestic	ICT (brand not reported)	Unspecified	Not reported in the available report; 6/31 reactive	6	31	19.4
Barbero-Moyano et al. (2024) (1)	Hunting seasons 2018-2019 to 2021-2022	Spain	*O. cuniculus*	Sylvatic	IFAT	IgG	≥1:50; clear cytoplasmic and membrane fluorescence in promastigotes	50	471	10.6

Study and host characteristics					Serological assessment			Raw prevalence outcome		
**Dataset**	**Sampling period**	**Country**	**Host species**	**Host status**	**Serological method**	**Antibody endpoint**	**Primary cut-off / positivity criterion**	**Seropositive (*n*)**	**Total examined (*n*)**	**Seroprevalence (%)**
Barbero-Moyano et al. (2024) (2)	Hunting seasons 2018-2019 to 2021-2022	Spain	*L. granatensis*	Sylvatic	IFAT	IgG	≥1:50; clear cytoplasmic and membrane fluorescence in promastigotes	31	160	19.4
Barbero-Moyano et al. (2025)	August 2022-April 2023	Spain	*O. cuniculus* and *L. granatensis*	Sylvatic	IFAT	Unspecified	Positive titre ≥1:50	19	41	46.3
Barbero-Moyano et al. (2026) (1)	2009-2012; three consecutive hunting seasons (August-July)	Spain	*O. cuniculus*	Sylvatic	IFAT	IgG	≥1:50	37	200	18.5
Barbero-Moyano et al. (2026) (2)	2012-2015; three consecutive hunting seasons (August-July)	Spain	*O. cuniculus*	Sylvatic	IFAT	IgG	≥1:50	33	218	15.1
Barbero-Moyano et al. (2026) (3)	2015-2018; three consecutive hunting seasons (August-July)	Spain	*O. cuniculus*	Sylvatic	IFAT	IgG	≥1:50	40	227	17.6
Barbero-Moyano et al. (2026) (4)	2018-2020; three consecutive hunting seasons (August-July)	Spain	*O. cuniculus*	Sylvatic	IFAT	IgG	≥1:50	23	214	10.7
Barbero-Moyano et al. (2026) (5)	2020-2023; three consecutive hunting seasons (August-July)	Spain	*O. cuniculus*	Sylvatic	IFAT	IgG	≥1:50	26	217	12.0

Notes: Raw positive and total counts correspond to the locked primary serological outcome used in the meta-analysis. When multiple titres or positivity thresholds were reported, the conservative positive criterion was used; doubtful, borderline, inconclusive, or lower-threshold-only reactions were not counted as positive.

The combined rabbit-hare dataset from Barbero-Moyano et al. (2025) was retained only in the overall lagomorph analysis because species-specific positive counts were not reported. Potential overlap among some Madrid datasets (García et al., 2014; Jiménez et al., 2014; Moreno et al., 2014; de la Cruz et al., 2016; Ortega et al., 2017) could not be excluded with certainty and was retained as a limitation of the evidence base. Silva, 2024 was retained as reported (6/31) despite limited methodological detail in the available report. Numbers in parentheses identify distinct eligible datasets derived from the same article.

Abbreviations: DAT, direct agglutination test; ELISA, enzyme-linked immunosorbent assay; EU, ELISA units; FITC, fluorescein isothiocyanate; IFAT, indirect fluorescent antibody test; ICT, immunochromatographic test; NR, not reported; OD, optical density; ROD, relative optical density; SD, standard deviation.

Supplementary Table S2B. Raw stratum-specific event and denominator data used in the random-effects meta-analyses of age- and sex-associated anti-*Leishmania* spp. seropositivity.

**Analysis unit**			**First comparison group**			**Second comparison group**			**Clarification**
**Factor**	**Study**	**Host population**	**Group 1**	**Positive (*n*)**	**Total (*n*)**	**Group 2**	**Positive (*n*)**	**Total (*n*)**	**Notes**
Age	García et al. (2014)	Rabbit (*O. cuniculus*)	Adult	20	26	Young	14	18	Age unavailable for 25 animals.
Age	de la Cruz et al. (2016)	Rabbits + hares combined	Adult	74	159	Young	26	94	Species-specific age counts were not reported; combined lagomorph counts were used.
Age	Ebani et al. (2016)	Hare (*L. europaeus*)	Adult	0	138	Juvenile	2	84	Juvenile animals were harmonized into the non-adult category.
Age	Barbero-Moyano et al. (2024)	Rabbits + hares combined	Adult	73	528	Non-adult	8	103	Non-adult = subadult 7/83 + young 1/20; species-specific age counts were not reported.
Sex	García et al. (2014)	Rabbit (*O. cuniculus*)	Male	13	16	Female	21	28	Sex unavailable for 25 animals.
Sex	Ebani et al. (2016)	Hare (*L. europaeus*)	Male	2	111	Female	0	111	Exact sex-specific event and denominator counts were reported.
Sex	Tsakmakidis et al. (2019) - rabbits	Rabbit (*O. cuniculus*); sylvatic + domestic combined	Male	23	240	Female	7	116	Rabbit sex results were reported across the combined rabbit populations; sex unknown for 37 rabbits.
Sex	Tsakmakidis et al. (2019) - hares	Hare (*L. europaeus*)	Male	3	51	Female	3	31	Sex unknown for 8 hares.
Sex	Barbero-Moyano et al. (2024)	Rabbits + hares combined	Male	36	309	Female	43	316	Species-specific sex counts were not reported; sex unavailable for 6 animals.

Notes: For the age analysis, adults were compared with a harmonized non-adult category. Young, juvenile, and subadult strata were classified as non-adult according to the terminology used in the original studies. In Barbero-Moyano et al. (2024), the non-adult count was derived by combining subadults (7/83) and young animals (1/20), yielding 8/103. For sex, males were compared with females. Only comparisons with exact positive and total counts for both strata were used. The Tsakmakidis et al. (2019) article contributed separate analyzable rabbit and hare populations. Counts shown here reproduce the data entered into the random-effects odds-ratio analyses reported in Table 3 of the main manuscript.
