# Supplementary material for: Serological evidence of Leishmania spp. exposure in rabbits and hares: A systematic review and meta-analysis
Source: Curr Res Parasitol Vector Borne Dis. 2026 Oct 27;10:100453. doi: 10.1016/j.crpvbd.2026.100453 (PMC13744300; doi:10.1016/j.crpvbd.2026.100453)
Supplement: Supplementary file 2 [file mmc2.pdf]

Supplementary file 2

Group by Publication year	Dataset	Statistics for each study: Event rate	Lower limit	Upper limit
<2019	Chitimia et al. (2011)	0.014	0.001	0.182
<2019	Díaz-Sáez et al. (2014)	0.204	0.147	0.277
<2019	García et al. (2014)	0.754	0.639	0.841
<2019	Jiménez et al. (2014)	0.229	0.132	0.368
<2019	Moreno et al. (2014) (1)	0.457	0.302	0.621
<2019	Moreno et al. (2014) (2)	0.741	0.638	0.823
<2019	Paiz et al. (2015)	0.125	0.017	0.537
<2019	de la Cruz et al. (2016) (1)	0.498	0.431	0.564
<2019	de la Cruz et al. (2016) (2)	0.174	0.102	0.282
<2019	Ebani et al. (2016)	0.009	0.002	0.035
<2019	Ortega et al. (2017) (1)	0.574	0.502	0.642
<2019	Ortega et al. (2017) (2)	0.213	0.128	0.333
<2019	Tsokana et al. (2019)	0.124	0.073	0.202
<2019	Tsakmakidis et al. (2019) (1)	0.010	0.001	0.067
<2019	Tsakmakidis et al. (2019) (2)	0.099	0.070	0.139
<2019	Tsakmakidis et al. (2019) (3)	0.067	0.030	0.141
<2019	Abbate et al. (2019)	0.025	0.004	0.157
<2019	Pooled	0.190	0.109	0.310
>2019	Athanasiou et al. (2021)	0.042	0.014	0.121
>2019	Cabezón et al. (2024) (1)	0.279	0.204	0.370
>2019	Cabezón et al. (2024) (2)	0.020	0.007	0.061
>2019	Silva et al. (2024)	0.194	0.090	0.369
>2019	Barbero-Moyano et al. (2024) (1)	0.106	0.081	0.137
>2019	Barbero-Moyano et al. (2024) (2)	0.194	0.140	0.262
>2019	Barbero-Moyano et al. (2025)	0.463	0.319	0.615
>2019	Barbero-Moyano et al. (2026) (1)	0.185	0.137	0.245
>2019	Barbero-Moyano et al. (2026) (2)	0.151	0.110	0.205
>2019	Barbero-Moyano et al. (2026) (3)	0.176	0.132	0.231
>2019	Barbero-Moyano et al. (2026) (4)	0.107	0.072	0.157
>2019	Barbero-Moyano et al. (2026) (5)	0.120	0.083	0.170
>2019	Pooled	0.153	0.113	0.203

Event rate and 95% CI

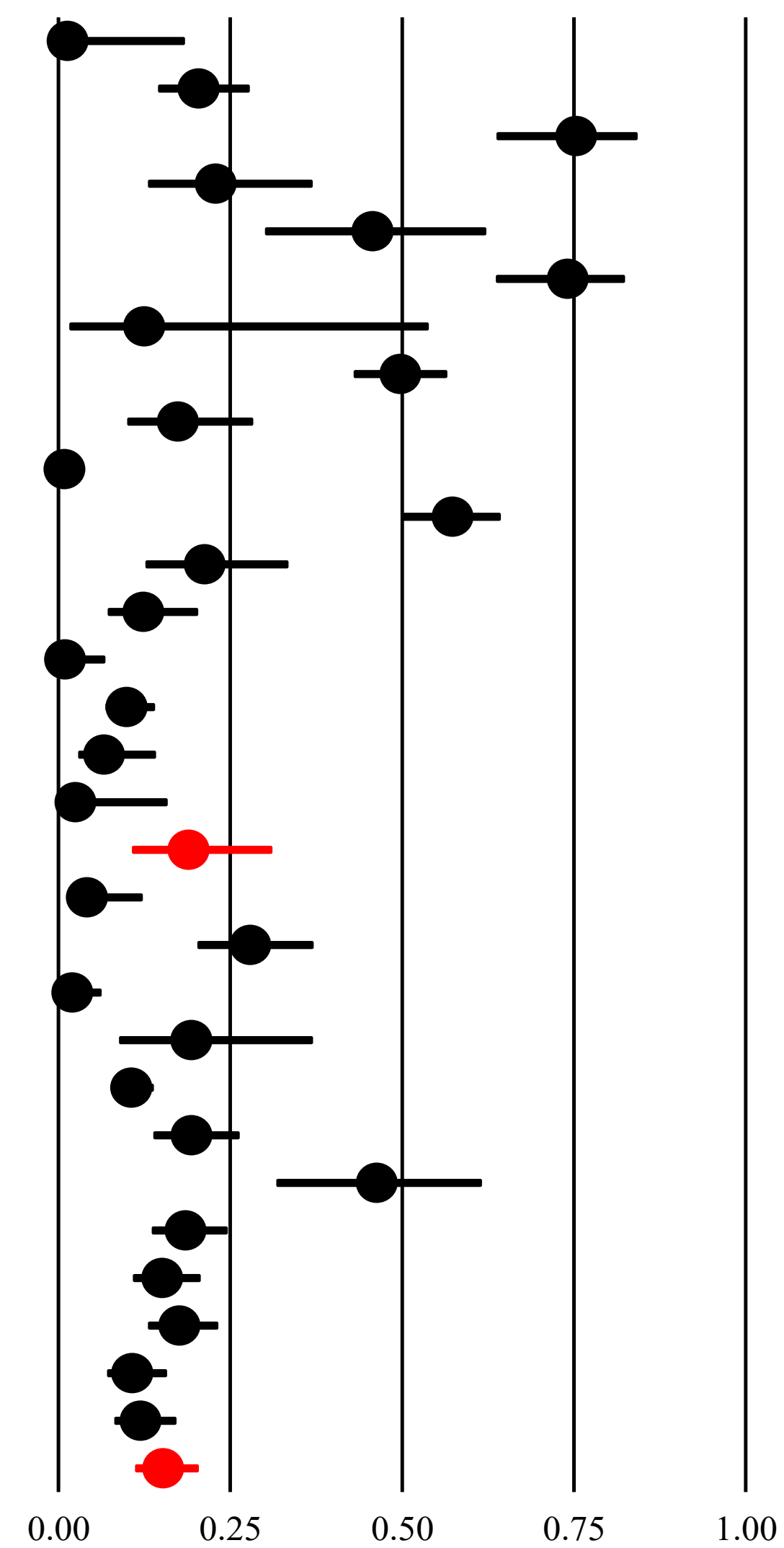

Supplementary Figure S1. The pooled seroprevalence of anti-*Leishmania* spp. antibodies in lagomorphs by publication year. Dataset numbers in parentheses following the study citation indicate distinct eligible datasets derived from the same article; the corresponding dataset labels and details are provided in Table 1. For graphical display, the “< 2019” label generated by CMA corresponds to the prespecified category of studies published in or before 2019 (≤ 2019).

Group by Sample size	Dataset	Statistics for each study: Event rate	Lower limit	Upper limit
<100	Chitimia et al. (2011)	0.014	0.001	0.182
<100	García et al. (2014)	0.754	0.639	0.841
<100	Jiménez et al. (2014)	0.229	0.132	0.368
<100	Moreno et al. (2014) (1)	0.457	0.302	0.621
<100	Moreno et al. (2014) (2)	0.741	0.638	0.823
<100	Paiz et al. (2015)	0.125	0.017	0.537
<100	de la Cruz et al. (2016) (2)	0.174	0.102	0.282
<100	Ortega et al. (2017) (2)	0.213	0.128	0.333
<100	Tsakmakidis et al. (2019) (3)	0.067	0.030	0.141
<100	Abbate et al. (2019)	0.025	0.004	0.157
<100	Athanasiou et al. (2021)	0.042	0.014	0.121
<100	Silva et al. (2024)	0.194	0.090	0.369
<100	Barbero-Moyano et al. (2025)	0.463	0.319	0.615
<100	Pooled	0.219	0.112	0.386
>100	Díaz-Sáez et al. (2014)	0.204	0.147	0.277
>100	de la Cruz et al. (2016) (1)	0.498	0.431	0.564
>100	Ebani et al. (2016)	0.009	0.002	0.035
>100	Ortega et al. (2017) (1)	0.574	0.502	0.642
>100	Tsokana et al. (2019)	0.124	0.073	0.202
>100	Tsakmakidis et al. (2019) (1)	0.010	0.001	0.067
>100	Tsakmakidis et al. (2019) (2)	0.099	0.070	0.139
>100	Cabezón et al. (2024) (1)	0.279	0.204	0.370
>100	Cabezón et al. (2024) (2)	0.020	0.007	0.061
>100	Barbero-Moyano et al. (2024) (1)	0.106	0.081	0.137
>100	Barbero-Moyano et al. (2024) (2)	0.194	0.140	0.262
>100	Barbero-Moyano et al. (2026) (1)	0.185	0.137	0.245
>100	Barbero-Moyano et al. (2026) (2)	0.151	0.110	0.205
>100	Barbero-Moyano et al. (2026) (3)	0.176	0.132	0.231
>100	Barbero-Moyano et al. (2026) (4)	0.107	0.072	0.157
>100	Barbero-Moyano et al. (2026) (5)	0.120	0.083	0.170
>100	Pooled	0.141	0.091	0.214

Event rate and 95% CI

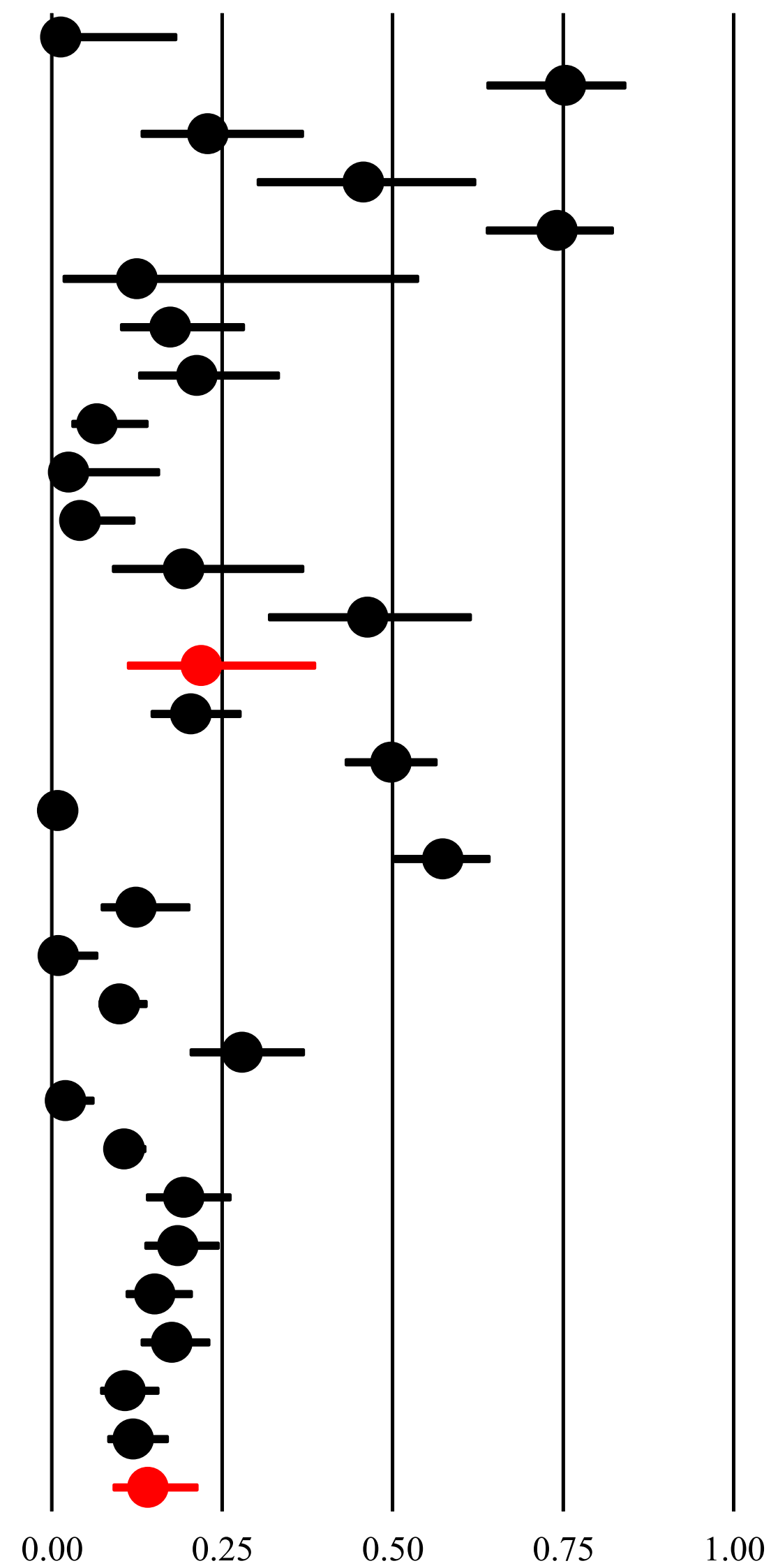

Supplementary Figure S2. The pooled seroprevalence of anti-*Leishmania* spp. antibodies in lagomorphs by sample size. Dataset numbers in parentheses following the study citation indicate distinct eligible datasets derived from the same article; the corresponding dataset labels and details are provided in Table 1. For graphical display, the "< 100" label generated by CMA corresponds to the prespecified category of datasets with a sample size of 100 or fewer (≤ 100).

Group by Country	Dataset	Statistics for each study: Event rate	Lower limit	Upper limit
Brazil	Paiz et al. (2015)	0.125	0.017	0.537
Brazil	Silva et al. (2024)	0.194	0.090	0.369
Brazil	Pooled	0.181	0.089	0.335
Greece	Tsokana et al. (2019)	0.124	0.073	0.202
Greece	Tsakmakidis et al. (2019) (1)	0.010	0.001	0.067
Greece	Tsakmakidis et al. (2019) (2)	0.099	0.070	0.139
Greece	Tsakmakidis et al. (2019) (3)	0.067	0.030	0.141
Greece	Athanasiou et al. (2021)	0.042	0.014	0.121
Greece	Pooled	0.076	0.046	0.122
Italy	Ebani et al. (2016)	0.009	0.002	0.035
Italy	Abbate et al. (2019)	0.025	0.004	0.157
Italy	Pooled	0.013	0.004	0.038
Spain	Chitimia et al. (2011)	0.014	0.001	0.182
Spain	Díaz-Sáez et al. (2014)	0.204	0.147	0.277
Spain	García et al. (2014)	0.754	0.639	0.841
Spain	Jiménez et al. (2014)	0.229	0.132	0.368
Spain	Moreno et al. (2014) (1)	0.457	0.302	0.621
Spain	Moreno et al. (2014) (2)	0.741	0.638	0.823
Spain	de la Cruz et al. (2016) (1)	0.498	0.431	0.564
Spain	de la Cruz et al. (2016) (2)	0.174	0.102	0.282
Spain	Ortega et al. (2017) (1)	0.574	0.502	0.642
Spain	Ortega et al. (2017) (2)	0.213	0.128	0.333
Spain	Cabezón et al. (2024) (1)	0.279	0.204	0.370
Spain	Cabezón et al. (2024) (2)	0.020	0.007	0.061
Spain	Barbero-Moyano et al. (2024) (1)	0.106	0.081	0.137
Spain	Barbero-Moyano et al. (2024) (2)	0.194	0.140	0.262
Spain	Barbero-Moyano et al. (2025)	0.463	0.319	0.615
Spain	Barbero-Moyano et al. (2026) (1)	0.185	0.137	0.245
Spain	Barbero-Moyano et al. (2026) (2)	0.151	0.110	0.205
Spain	Barbero-Moyano et al. (2026) (3)	0.176	0.132	0.231
Spain	Barbero-Moyano et al. (2026) (4)	0.107	0.072	0.157
Spain	Barbero-Moyano et al. (2026) (5)	0.120	0.083	0.170
Spain	Pooled	0.246	0.169	0.345

Event rate and 95% CI

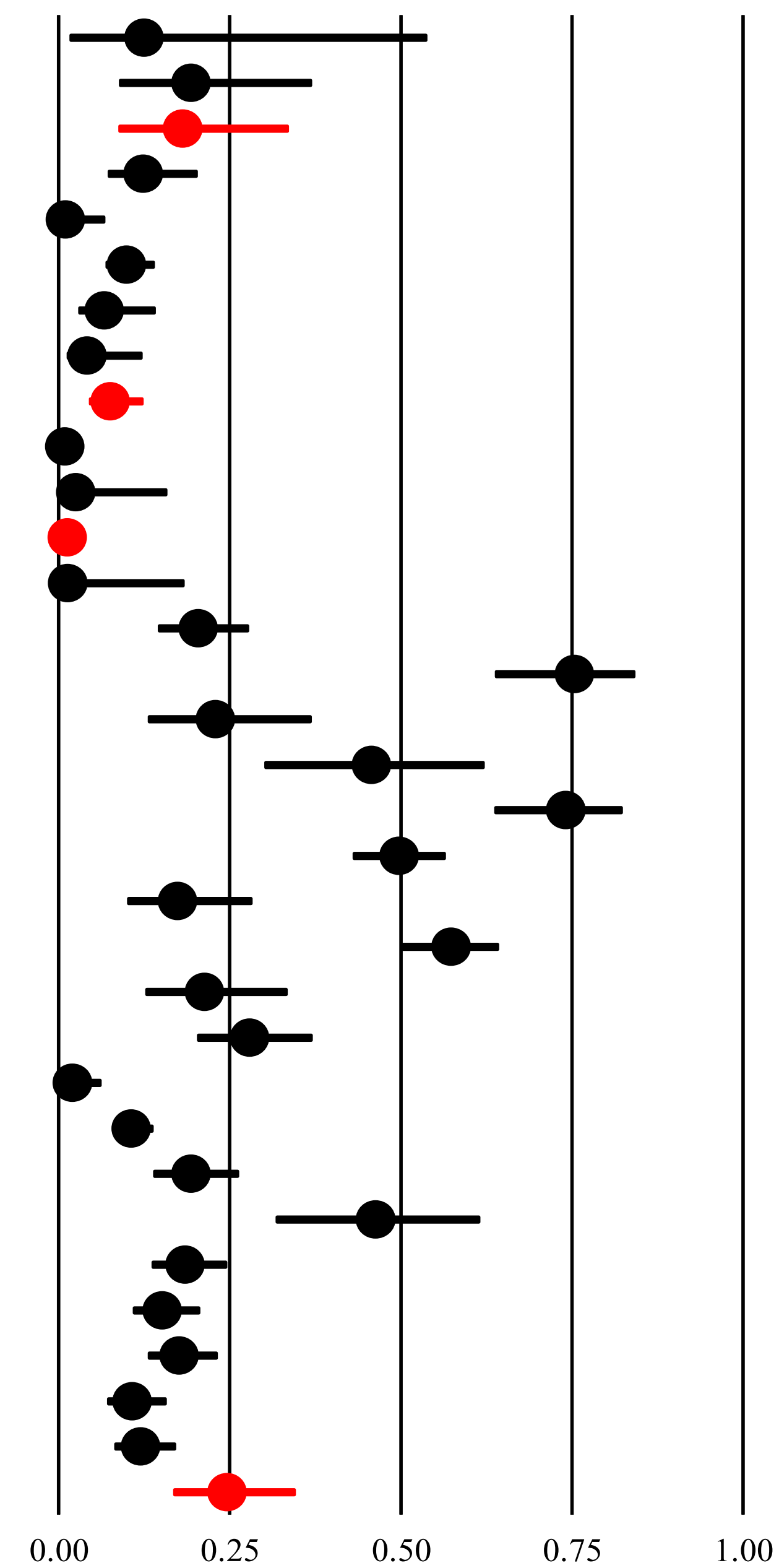

Supplementary Figure S3. The pooled seroprevalence of anti-*Leishmania* spp. antibodies in lagomorphs by country. Dataset numbers in parentheses following the study citation indicate distinct eligible datasets derived from the same article; the corresponding dataset labels and details are provided in Table 1.

Group by Host type	Dataset	Statistics for each study: Event rate	Lower limit	Upper limit
Hare	Moreno et al. (2014) (2)	0.741	0.638	0.823
Hare	Paiz et al. (2015)	0.125	0.017	0.537
Hare	de la Cruz et al. (2016) (2)	0.174	0.102	0.282
Hare	Ebani et al. (2016)	0.009	0.002	0.035
Hare	Ortega et al. (2017) (2)	0.213	0.128	0.333
Hare	Tsokana et al. (2019)	0.124	0.073	0.202
Hare	Tsakmakidis et al. (2019) (3)	0.067	0.030	0.141
Hare	Cabezón et al. (2024) (2)	0.020	0.007	0.061
Hare	Barbero-Moyano et al. (2024) (2)	0.194	0.140	0.262
Hare	Pooled	0.124	0.050	0.274
Rabbit	Chitimia et al. (2011)	0.014	0.001	0.182
Rabbit	Díaz-Sáez et al. (2014)	0.204	0.147	0.277
Rabbit	García et al. (2014)	0.754	0.639	0.841
Rabbit	Jiménez et al. (2014)	0.229	0.132	0.368
Rabbit	Moreno et al. (2014) (1)	0.457	0.302	0.621
Rabbit	de la Cruz et al. (2016) (1)	0.498	0.431	0.564
Rabbit	Ortega et al. (2017) (1)	0.574	0.502	0.642
Rabbit	Tsakmakidis et al. (2019) (1)	0.010	0.001	0.067
Rabbit	Tsakmakidis et al. (2019) (2)	0.099	0.070	0.139
Rabbit	Abbate et al. (2019)	0.025	0.004	0.157
Rabbit	Athanasiou et al. (2021)	0.042	0.014	0.121
Rabbit	Cabezón et al. (2024) (1)	0.279	0.204	0.370
Rabbit	Silva et al. (2024)	0.194	0.090	0.369
Rabbit	Barbero-Moyano et al. (2024) (1)	0.106	0.081	0.137
Rabbit	Barbero-Moyano et al. (2026) (1)	0.185	0.137	0.245
Rabbit	Barbero-Moyano et al. (2026) (2)	0.151	0.110	0.205
Rabbit	Barbero-Moyano et al. (2026) (3)	0.176	0.132	0.231
Rabbit	Barbero-Moyano et al. (2026) (4)	0.107	0.072	0.157
Rabbit	Barbero-Moyano et al. (2026) (5)	0.120	0.083	0.170
Rabbit	Pooled	0.186	0.121	0.273

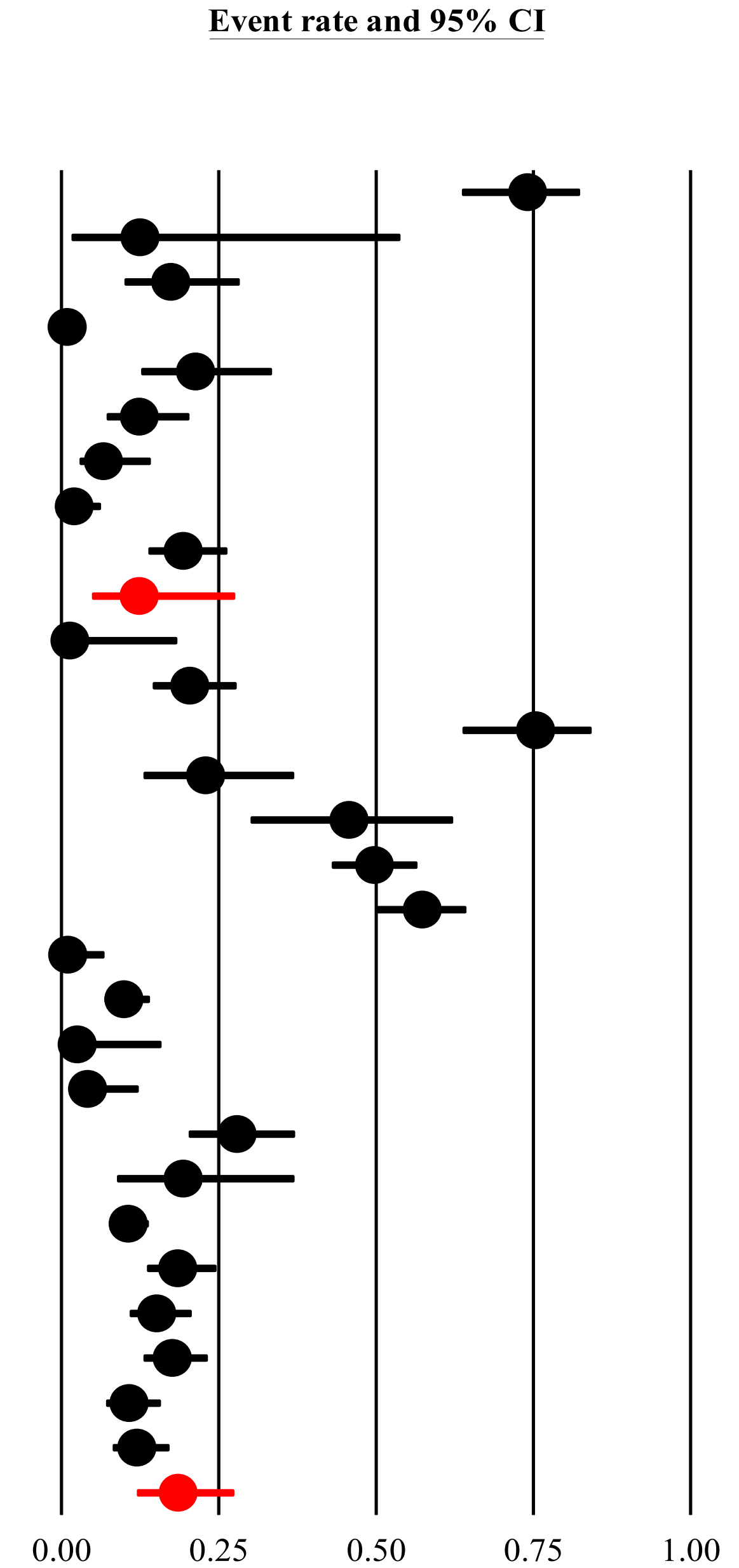

Supplementary Figure S4. The pooled seroprevalence of anti-*Leishmania* spp. antibodies in lagomorphs by host type. Dataset numbers in parentheses following the study citation indicate distinct eligible datasets derived from the same article; the corresponding dataset labels and details are provided in Table 1.

Group by Methods	Dataset	Statistics for each study: Event rate	Lower limit	Upper limit
Agglutination test	Paiz et al. (2015)	0.125	0.017	0.537
Agglutination test	Pooled	0.125	0.017	0.537
ELISA	Chitimia et al. (2011)	0.014	0.001	0.182
ELISA	Tsakmakidis et al. (2019) (1)	0.010	0.001	0.067
ELISA	Tsakmakidis et al. (2019) (2)	0.099	0.070	0.139
ELISA	Tsakmakidis et al. (2019) (3)	0.067	0.030	0.141
ELISA	Cabezón et al. (2024) (1)	0.279	0.204	0.370
ELISA	Cabezón et al. (2024) (2)	0.020	0.007	0.061
ELISA	Pooled	0.060	0.023	0.145
IFAT	Díaz-Sáez et al. (2014)	0.204	0.147	0.277
IFAT	García et al. (2014)	0.754	0.639	0.841
IFAT	Moreno et al. (2014) (1)	0.457	0.302	0.621
IFAT	Moreno et al. (2014) (2)	0.741	0.638	0.823
IFAT	de la Cruz et al. (2016) (1)	0.498	0.431	0.564
IFAT	de la Cruz et al. (2016) (2)	0.174	0.102	0.282
IFAT	Ebani et al. (2016)	0.009	0.002	0.035
IFAT	Ortega et al. (2017) (1)	0.574	0.502	0.642
IFAT	Ortega et al. (2017) (2)	0.213	0.128	0.333
IFAT	Tsokana et al. (2019)	0.124	0.073	0.202
IFAT	Athanasiou et al. (2021)	0.042	0.014	0.121
IFAT	Barbero-Moyano et al. (2024) (1)	0.106	0.081	0.137
IFAT	Barbero-Moyano et al. (2024) (2)	0.194	0.140	0.262
IFAT	Barbero-Moyano et al. (2025)	0.463	0.319	0.615
IFAT	Barbero-Moyano et al. (2026) (1)	0.185	0.137	0.245
IFAT	Barbero-Moyano et al. (2026) (2)	0.151	0.110	0.205
IFAT	Barbero-Moyano et al. (2026) (3)	0.176	0.132	0.231
IFAT	Barbero-Moyano et al. (2026) (4)	0.107	0.072	0.157
IFAT	Barbero-Moyano et al. (2026) (5)	0.120	0.083	0.170
IFAT	Pooled	0.229	0.152	0.330
Immunochromatographic/Rapid test	Jiménez et al. (2014)	0.229	0.132	0.368
Immunochromatographic/Rapid test	Abbate et al. (2019)	0.025	0.004	0.157
Immunochromatographic/Rapid test	Silva et al. (2024)	0.194	0.090	0.369
Immunochromatographic/Rapid test	Pooled	0.153	0.064	0.324

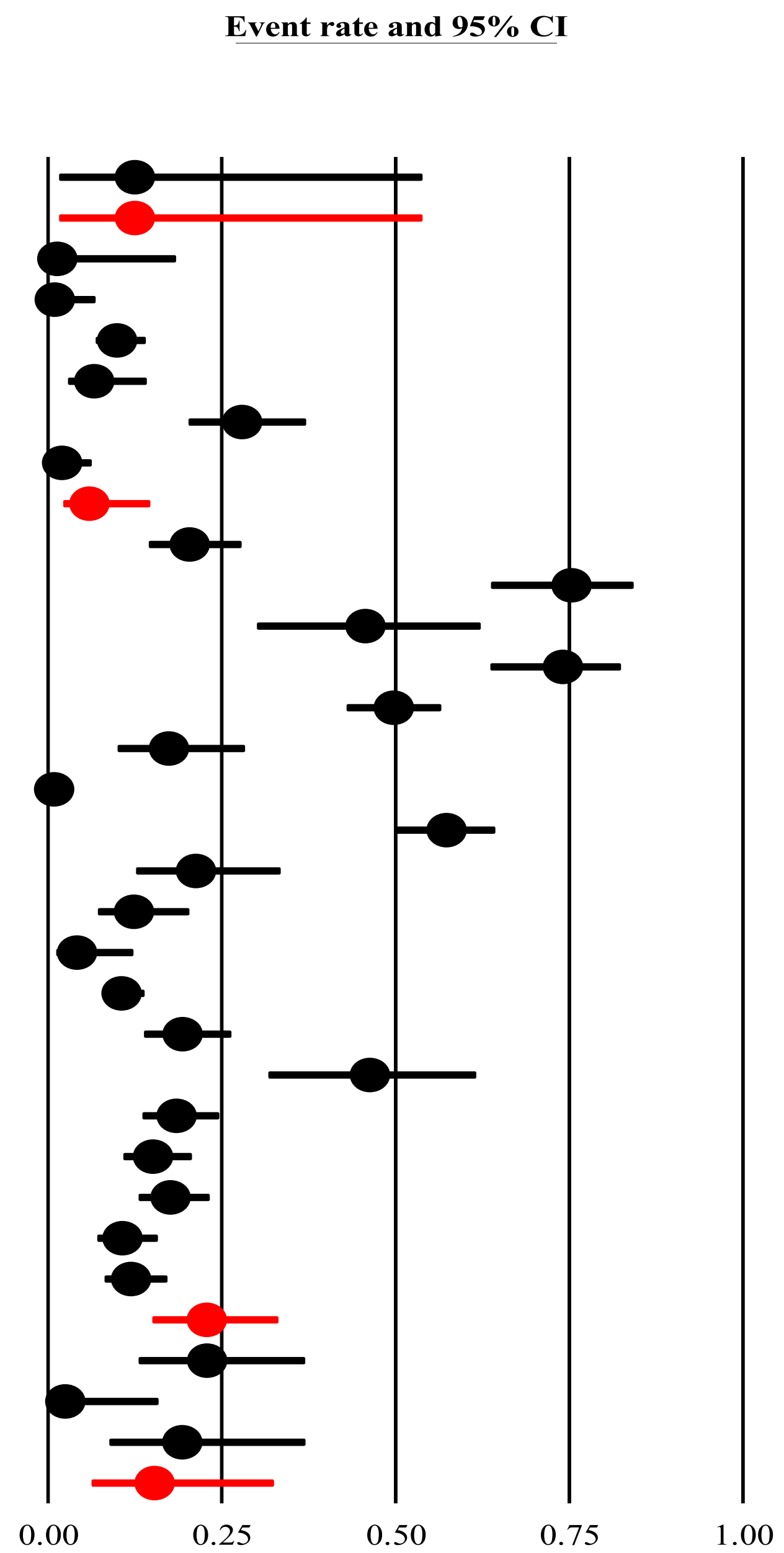

Supplementary Figure S5. The pooled seroprevalence of anti-*Leishmania* spp. antibodies in lagomorphs by serological method. Dataset numbers in parentheses following the study citation indicate distinct eligible datasets derived from the same article; the corresponding dataset labels and details are provided in Table 1.

	Dataset	Statistics for each study			Event rate and 95% CI
		Event rate	Lower limit	Upper limit	
IgG only	Chitimia et al. (2011)	0.014	0.001	0.182	
IgG only	Ebani et al. (2016)	0.009	0.002	0.035	
IgG only	Tsokana et al. (2019)	0.124	0.073	0.202	
IgG only	Tsakmakidis et al. (2019) (1)	0.010	0.001	0.067	
IgG only	Tsakmakidis et al. (2019) (2)	0.099	0.070	0.139	
IgG only	Tsakmakidis et al. (2019) (3)	0.067	0.030	0.141	
IgG only	Athanasiou et al. (2021)	0.042	0.014	0.121	
IgG only	Barbero-Moyano et al. (2024) (1)	0.106	0.081	0.137	
IgG only	Barbero-Moyano et al. (2024) (2)	0.194	0.140	0.262	
IgG only	Barbero-Moyano et al. (2026) (1)	0.185	0.137	0.245	
IgG only	Barbero-Moyano et al. (2026) (2)	0.151	0.110	0.205	
IgG only	Barbero-Moyano et al. (2026) (3)	0.176	0.132	0.231	
IgG only	Barbero-Moyano et al. (2026) (4)	0.107	0.072	0.157	
IgG only	Barbero-Moyano et al. (2026) (5)	0.120	0.083	0.170	
IgG only	Pooled	0.109	0.084	0.140	
Total/Not differentiated	Díaz-Sáez et al. (2014)	0.204	0.147	0.277	
Total/Not differentiated	García et al. (2014)	0.754	0.639	0.841	
Total/Not differentiated	Jiménez et al. (2014)	0.229	0.132	0.368	
Total/Not differentiated	Paiz et al. (2015)	0.125	0.017	0.537	
Total/Not differentiated	de la Cruz et al. (2016) (1)	0.498	0.431	0.564	
Total/Not differentiated	de la Cruz et al. (2016) (2)	0.174	0.102	0.282	
Total/Not differentiated	Ortega et al. (2017) (1)	0.574	0.502	0.642	
Total/Not differentiated	Ortega et al. (2017) (2)	0.213	0.128	0.333	
Total/Not differentiated	Abbate et al. (2019)	0.025	0.004	0.157	
Total/Not differentiated	Cabezón et al. (2024) (1)	0.279	0.204	0.370	
Total/Not differentiated	Cabezón et al. (2024) (2)	0.020	0.007	0.061	
Total/Not differentiated	Pooled	0.250	0.150	0.386	
Unspecified antibody endpoint	Moreno et al. (2014) (1)	0.457	0.302	0.621	
Unspecified antibody endpoint	Moreno et al. (2014) (2)	0.741	0.638	0.823	
Unspecified antibody endpoint	Silva et al. (2024)	0.194	0.090	0.369	
Unspecified antibody endpoint	Barbero-Moyano et al. (2025)	0.463	0.319	0.615	
Unspecified antibody endpoint	Pooled	0.468	0.251	0.698	

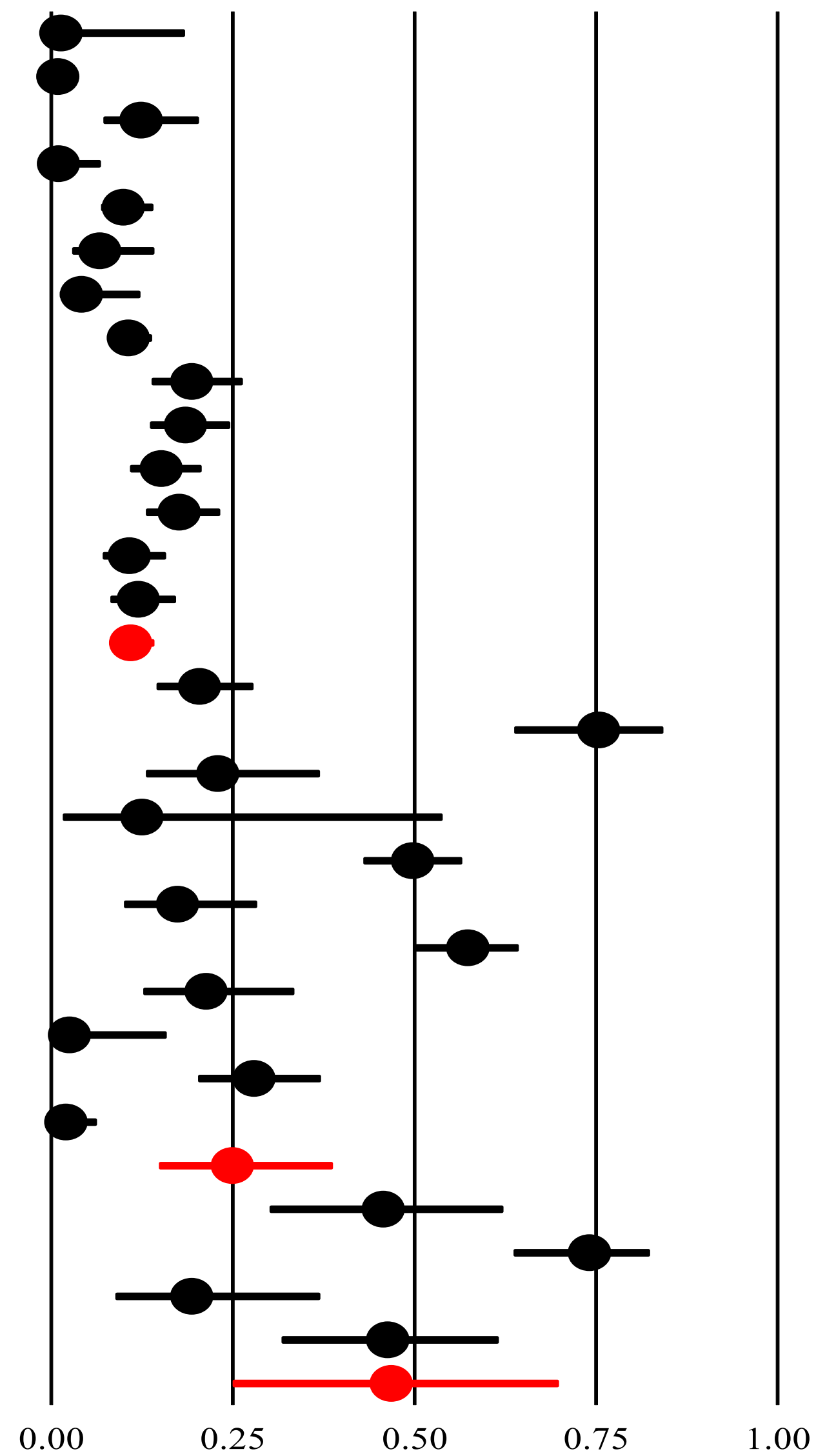

Supplementary Figure S6. The pooled seroprevalence of anti-*Leishmania* spp. antibodies in lagomorphs by antibody endpoint. Dataset numbers in parentheses following the study citation indicate distinct eligible datasets derived from the same article; the corresponding dataset labels and details are provided in Table 1.

Group by Rabbit type	Dataset	Event rate	Lower limit	Upper limit
Domestic	Tsakmakidis et al. (2019) (2)	0.099	0.070	0.139
Domestic	Abbate et al. (2019)	0.025	0.004	0.157
Domestic	Silva et al. (2024)	0.194	0.090	0.369
Domestic	Pooled	0.107	0.050	0.212
Sylvatic	Chitimia et al. (2011)	0.014	0.001	0.182
Sylvatic	Díaz-Sáez et al. (2014)	0.204	0.147	0.277
Sylvatic	García et al. (2014)	0.754	0.639	0.841
Sylvatic	Jiménez et al. (2014)	0.229	0.132	0.368
Sylvatic	Moreno et al. (2014) (1)	0.457	0.302	0.621
Sylvatic	de la Cruz et al. (2016) (1)	0.498	0.431	0.564
Sylvatic	Ortega et al. (2017) (1)	0.574	0.502	0.642
Sylvatic	Tsakmakidis et al. (2019) (1)	0.010	0.001	0.067
Sylvatic	Athanasiou et al. (2021)	0.042	0.014	0.121
Sylvatic	Cabezón et al. (2024) (1)	0.279	0.204	0.370
Sylvatic	Barbero-Moyano et al. (2024) (1)	0.106	0.081	0.137
Sylvatic	Barbero-Moyano et al. (2026) (1)	0.185	0.137	0.245
Sylvatic	Barbero-Moyano et al. (2026) (2)	0.151	0.110	0.205
Sylvatic	Barbero-Moyano et al. (2026) (3)	0.176	0.132	0.231
Sylvatic	Barbero-Moyano et al. (2026) (4)	0.107	0.072	0.157
Sylvatic	Barbero-Moyano et al. (2026) (5)	0.120	0.083	0.170
Sylvatic	Pooled	0.205	0.131	0.308

Statistics for each study

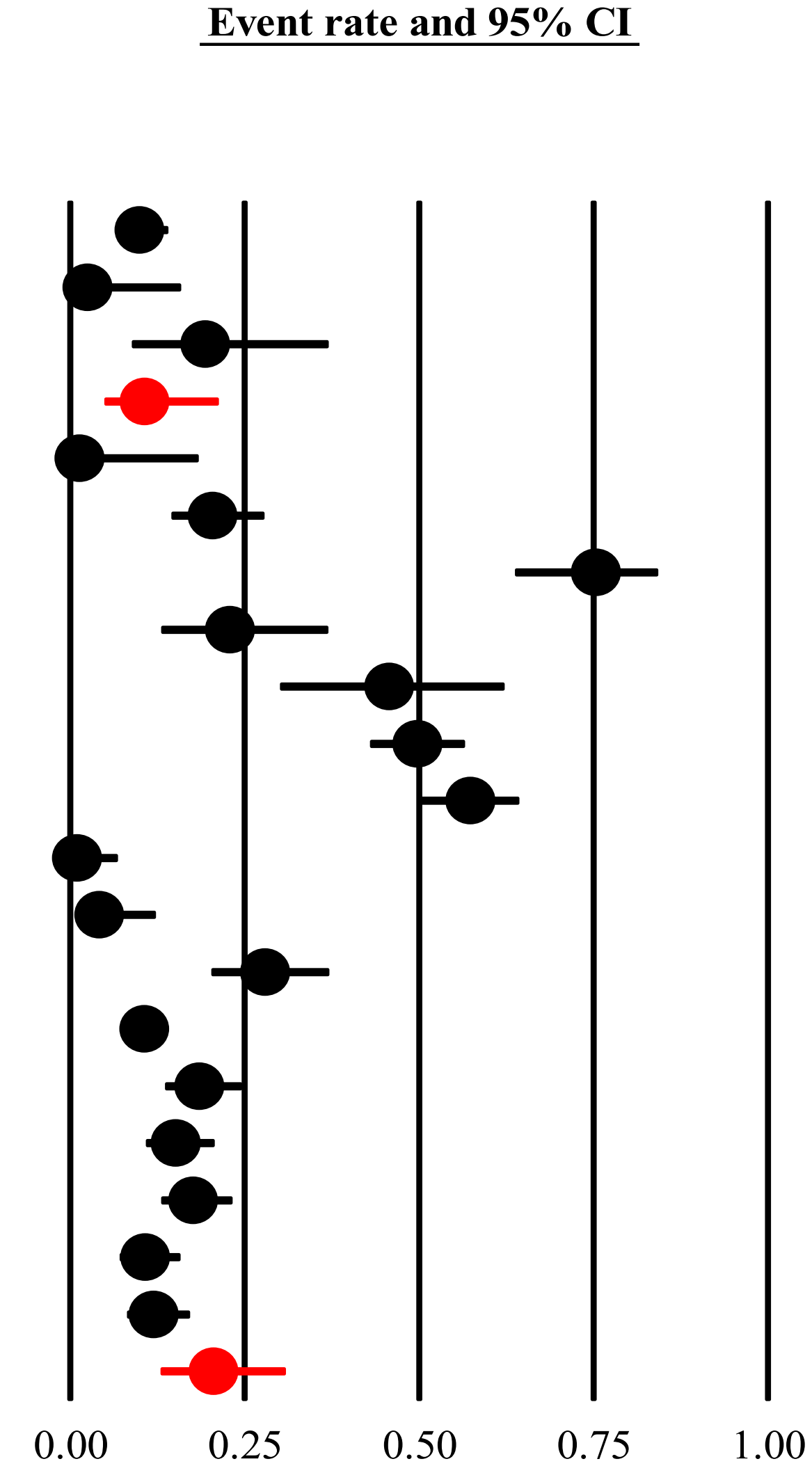

Supplementary Figure S7. The pooled seroprevalence of anti-*Leishmania* spp. antibodies in rabbits by rabbit type. Dataset numbers in parentheses following the study citation indicate distinct eligible datasets derived from the same article; the corresponding dataset labels and details are provided in Table 1.

**Group by Hare species**	**Study name**	**Statistics for each study**			**Event rate and 95% CI**
		Event rate	Lower limit	Upper limit	
Lepus europaeus	Paiz et al. (2015)	0.125	0.017	0.537	
Lepus europaeus	Ebani et al. (2016)	0.009	0.002	0.035	
Lepus europaeus	Tsokana et al. (2019)	0.124	0.073	0.202	
Lepus europaeus	Tsakmakidis et al. (2019) (3)	0.067	0.030	0.141	
Lepus europaeus	Cabezón et al. (2024) (2)	0.020	0.007	0.061	
Lepus europaeus	Pooled	0.047	0.018	0.120	
Lepus granatensis	Moreno et al. (2014) (2)	0.741	0.638	0.823	
Lepus granatensis	de la Cruz et al. (2016) (2)	0.174	0.102	0.282	
Lepus granatensis	Ortega et al. (2017) (2)	0.213	0.128	0.333	
Lepus granatensis	Barbero-Moyano et al. (2024) (2)	0.194	0.140	0.262	
Lepus granatensis	Pooled	0.309	0.111	0.616	

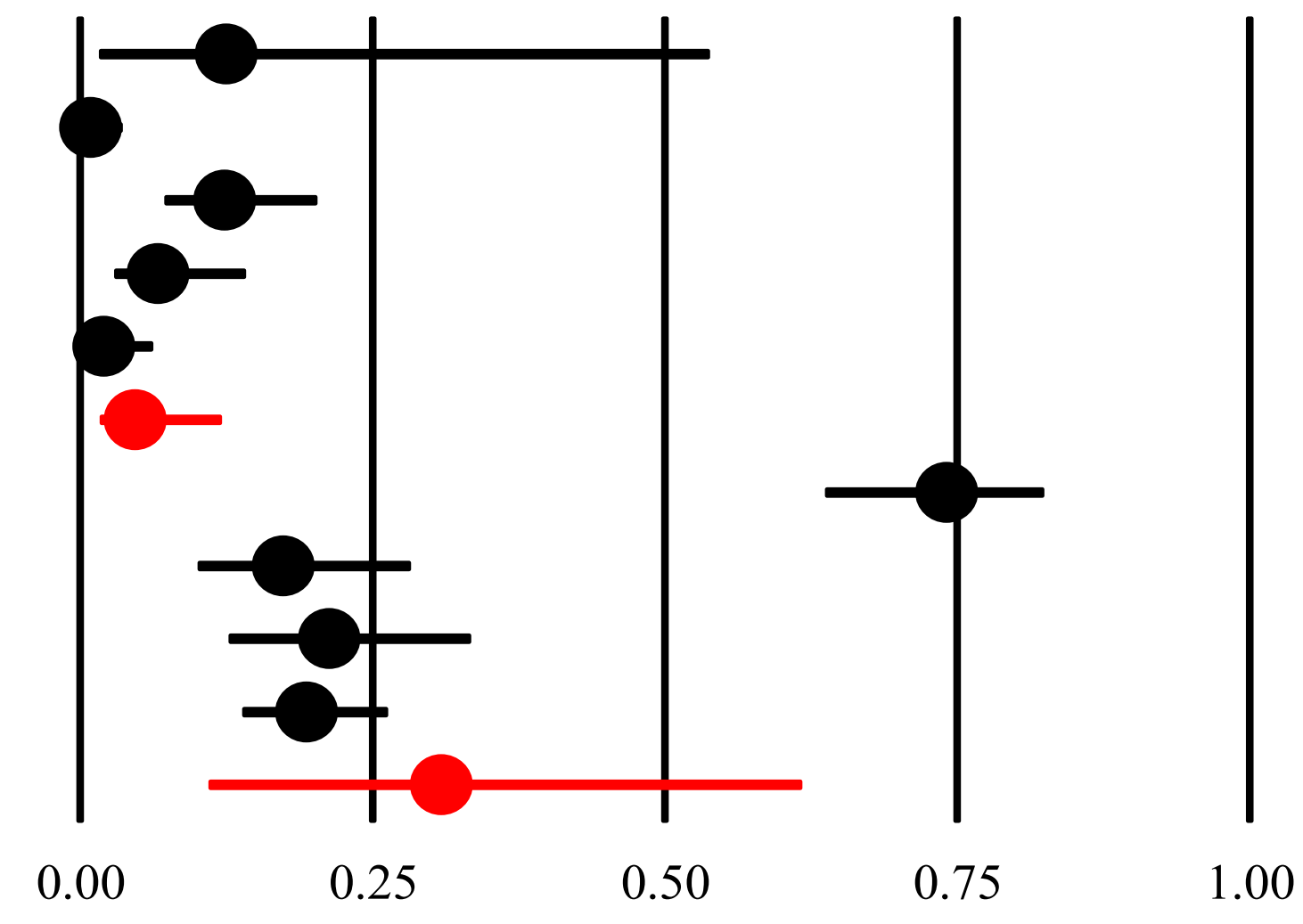

Supplementary Figure S8. The pooled seroprevalence of anti-*Leishmania* spp. antibodies in hares by hare species. Dataset numbers in parentheses following the study citation indicate distinct eligible datasets derived from the same article; the corresponding dataset labels and details are provided in Table 1.

Study name	Statistics for each study					Exposed / Total	
	Odds ratio	Lower limit	Upper limit	Z-Value	p-Value	Adult	Non-adult
de la Cruz et al. (2016)	2.277	1.315	3.942	2.938	0.003	74 / 159	26 / 94
Ebani et al. (2016)	0.119	0.006	2.512	-1.368	0.171	0 / 138	2 / 84
García et al. (2014)	0.952	0.226	4.011	-0.067	0.947	20 / 26	14 / 18
Barbero-Moyano et al. (2024)	1.905	0.889	4.085	1.657	0.098	73 / 528	8 / 103
Pooled	1.690	0.923	3.096	1.700	0.089		

Odds ratio and 95% CI

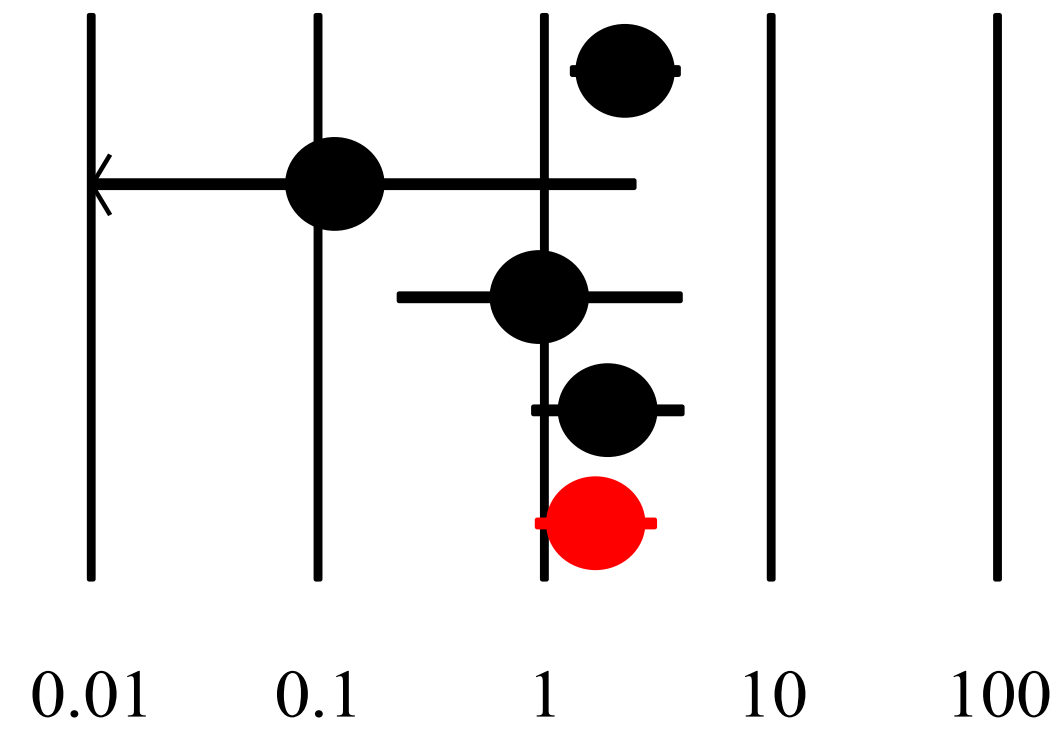

Supplementary Figure S9. Forest plot of the association between age and anti-*Leishmania* spp. seropositivity in lagomorphs.

Dataset	Statistics for each study				Exposed / Total		Odds ratio and 95% CI
	Odds ratio	Lower limit	Upper limit	p-Value	Male	Female	
Tsakmakidis et al. (2019) (1)	1.650	0.687	3.966	0.263	23 / 240	7 / 116	
Tsakmakidis et al. (2019) (2)	0.583	0.110	3.089	0.526	3 / 51	3 / 31	
Ebani et al. (2016)	5.091	0.242	107.268	0.295	2 / 111	0 / 111	
García et al. (2014)	1.444	0.316	6.598	0.635	13 / 16	21 / 28	
Barbero-Moyano et al. (2024)	0.837	0.521	1.344	0.462	36 / 309	43 / 316	
Pooled	1.000	0.679	1.473	1.000			

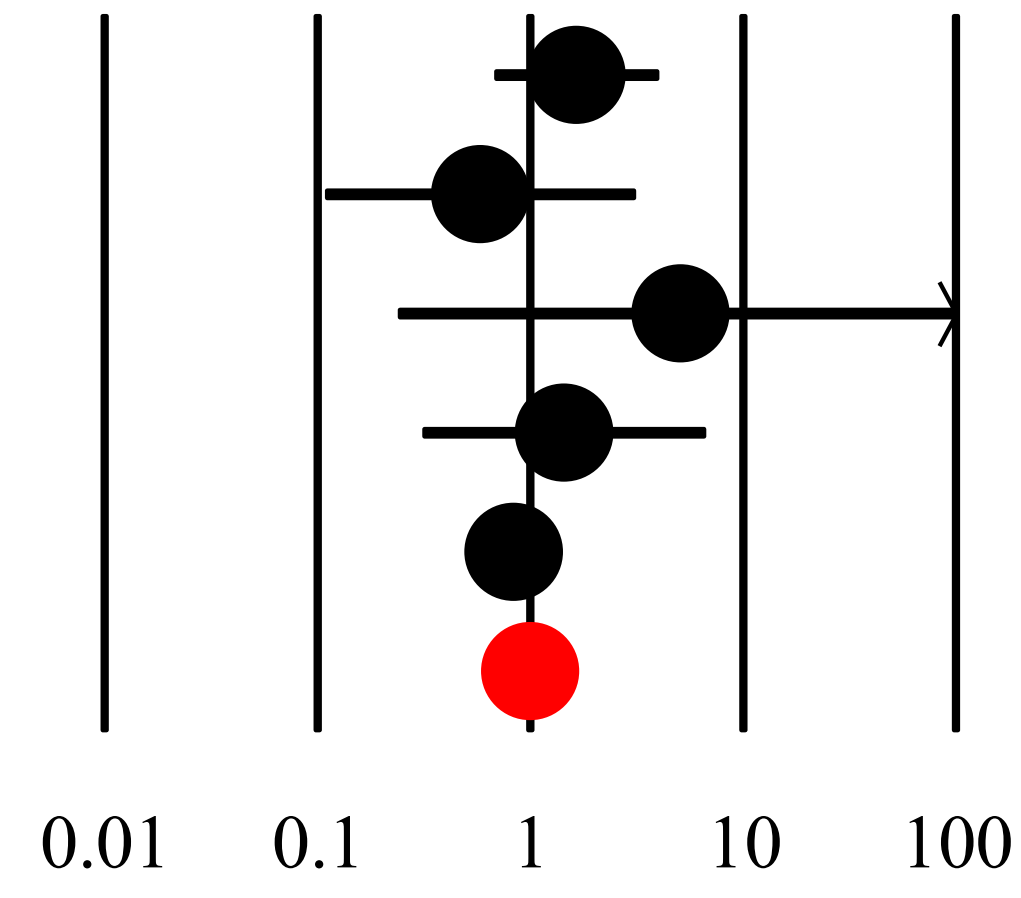

Supplementary Figure S10. Forest plot of the association between sex and anti-*Leishmania* spp. seropositivity in lagomorphs. Dataset numbers in parentheses following the study citation indicate distinct eligible datasets derived from the same article; the corresponding dataset labels and details are provided in Table 1.

Dataset	Statistics with study removed		
	Point	Lower limit	Upper limit
Chitimia et al. (2011)	0.179	0.124	0.250
Díaz-Sáez et al. (2014)	0.171	0.117	0.243
García et al. (2014)	0.160	0.112	0.223
Jiménez et al. (2014)	0.170	0.117	0.241
Moreno et al. (2014) (1)	0.165	0.114	0.234
Moreno et al. (2014) (2)	0.160	0.113	0.223
Paiz et al. (2015)	0.174	0.120	0.244
de la Cruz et al. (2016) (1)	0.164	0.114	0.231
de la Cruz et al. (2016) (2)	0.172	0.118	0.244
Ebani et al. (2016)	0.187	0.131	0.259
Ortega et al. (2017) (1)	0.164	0.115	0.228
Ortega et al. (2017) (2)	0.171	0.117	0.242
Tsokana et al. (2019)	0.175	0.120	0.246
Tsakmakidis et al. (2019) (1)	0.183	0.127	0.255
Tsakmakidis et al. (2019) (2)	0.176	0.122	0.248
Tsakmakidis et al. (2019) (3)	0.178	0.124	0.250
Abbate et al. (2019)	0.179	0.125	0.251
Athanasiou et al. (2021)	0.180	0.125	0.252
Cabezón et al. (2024) (1)	0.168	0.115	0.240
Cabezón et al. (2024) (2)	0.184	0.128	0.256
Silva et al. (2024)	0.172	0.118	0.242
Barbero-Moyano et al. (2024) (1)	0.176	0.121	0.248
Barbero-Moyano et al. (2024) (2)	0.171	0.117	0.243
Barbero-Moyano et al. (2025)	0.165	0.113	0.234
Barbero-Moyano et al. (2026) (1)	0.171	0.117	0.244
Barbero-Moyano et al. (2026) (2)	0.173	0.118	0.245
Barbero-Moyano et al. (2026) (3)	0.171	0.117	0.244
Barbero-Moyano et al. (2026) (4)	0.176	0.121	0.248
Barbero-Moyano et al. (2026) (5)	0.175	0.120	0.247
Pooled	0.173	0.120	0.242

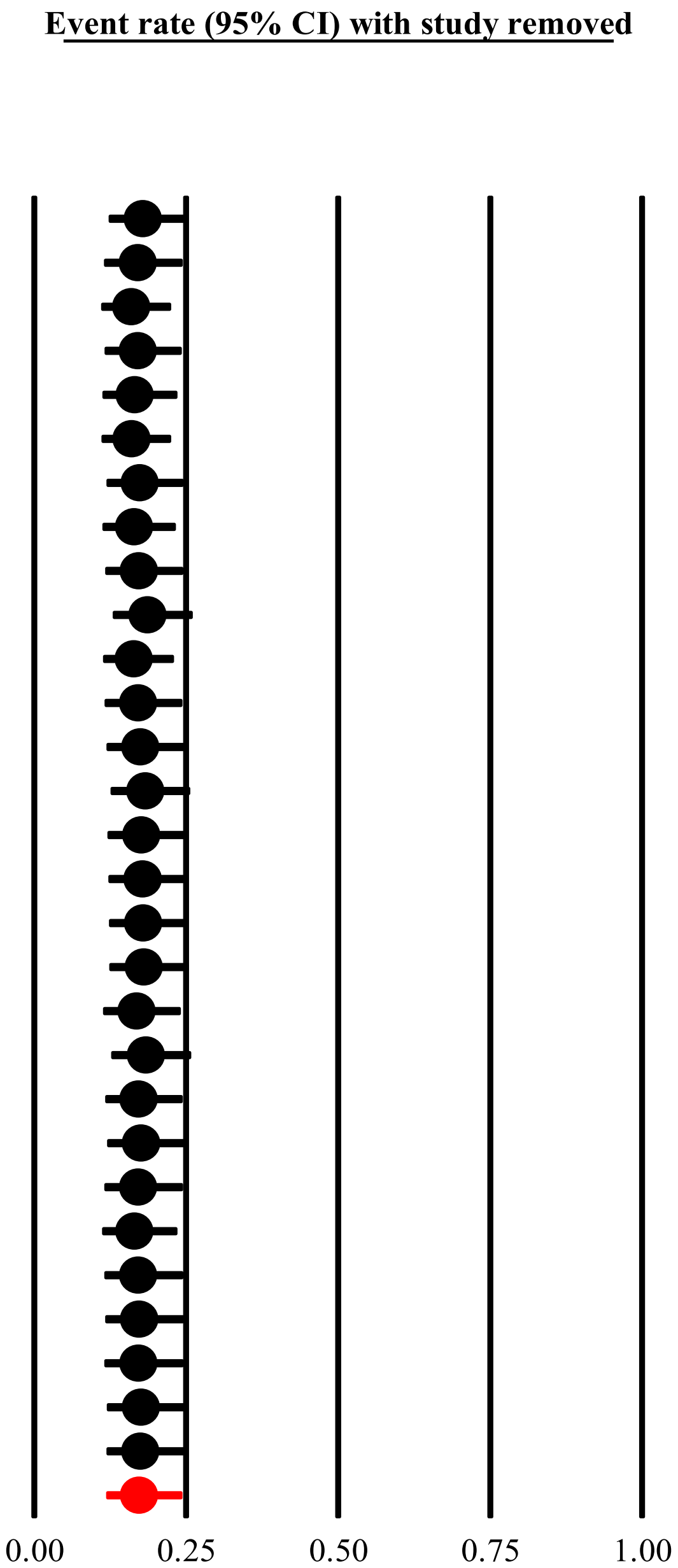

Supplementary Figure S11. Sensitivity analysis assessing the influence of individual studies on pooled estimates. Dataset numbers in parentheses following the study citation indicate distinct eligible datasets derived from the same article; the corresponding dataset labels and details are provided in Table 1.

Dataset	Statistics with study removed			Event rate (95% CI) with study removed
	Point	Lower limit	Upper limit	
Chitimia et al. (2011)	0.180	0.125	0.252	
Díaz-Sáez et al. (2014)	0.172	0.117	0.245	
García et al. (2014)	0.161	0.112	0.225	
Jiménez et al. (2014)	0.171	0.117	0.244	
Moreno et al. (2014) (1)	0.166	0.114	0.236	
Moreno et al. (2014) (2)	0.161	0.113	0.225	
de la Cruz et al. (2016) (1)	0.165	0.114	0.233	
de la Cruz et al. (2016) (2)	0.173	0.119	0.246	
Ebani et al. (2016)	0.188	0.131	0.262	
Ortega et al. (2017) (1)	0.165	0.115	0.230	
Ortega et al. (2017) (2)	0.172	0.118	0.244	
Tsokana et al. (2019)	0.176	0.121	0.249	
Tsakmakidis et al. (2019) (1)	0.184	0.128	0.258	
Tsakmakidis et al. (2019) (2)	0.178	0.122	0.251	
Tsakmakidis et al. (2019) (3)	0.180	0.124	0.253	
Abbate et al. (2019)	0.181	0.125	0.254	
Athanasiou et al. (2021)	0.181	0.126	0.255	
Cabezón et al. (2024) (1)	0.169	0.115	0.242	
Cabezón et al. (2024) (2)	0.185	0.129	0.259	
Silva et al. (2024)	0.173	0.119	0.245	
Barbero-Moyano et al. (2024) (1)	0.177	0.122	0.250	
Barbero-Moyano et al. (2024) (2)	0.172	0.117	0.246	
Barbero-Moyano et al. (2025)	0.166	0.114	0.236	
Barbero-Moyano et al. (2026) (1)	0.172	0.117	0.246	
Barbero-Moyano et al. (2026) (2)	0.174	0.119	0.248	
Barbero-Moyano et al. (2026) (3)	0.172	0.117	0.247	
Barbero-Moyano et al. (2026) (4)	0.177	0.121	0.250	
Barbero-Moyano et al. (2026) (5)	0.176	0.121	0.249	
Pooled	0.174	0.120	0.244	

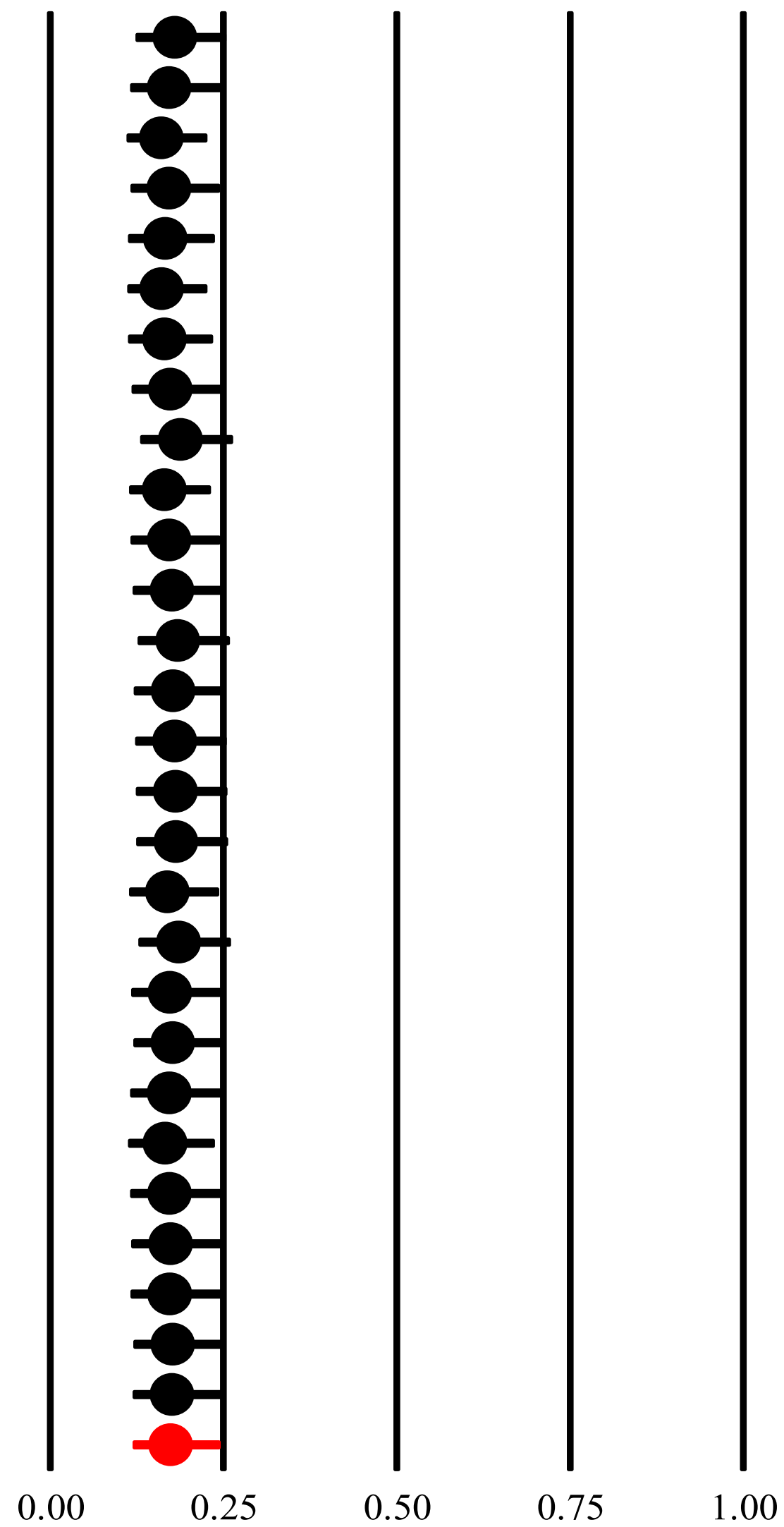

Supplementary Figure S12. Sensitivity analysis excluding dataset with fewer than 10 samples. Dataset numbers in parentheses following the study citation indicate distinct eligible datasets derived from the same article; the corresponding dataset labels and details are provided in Table 1.

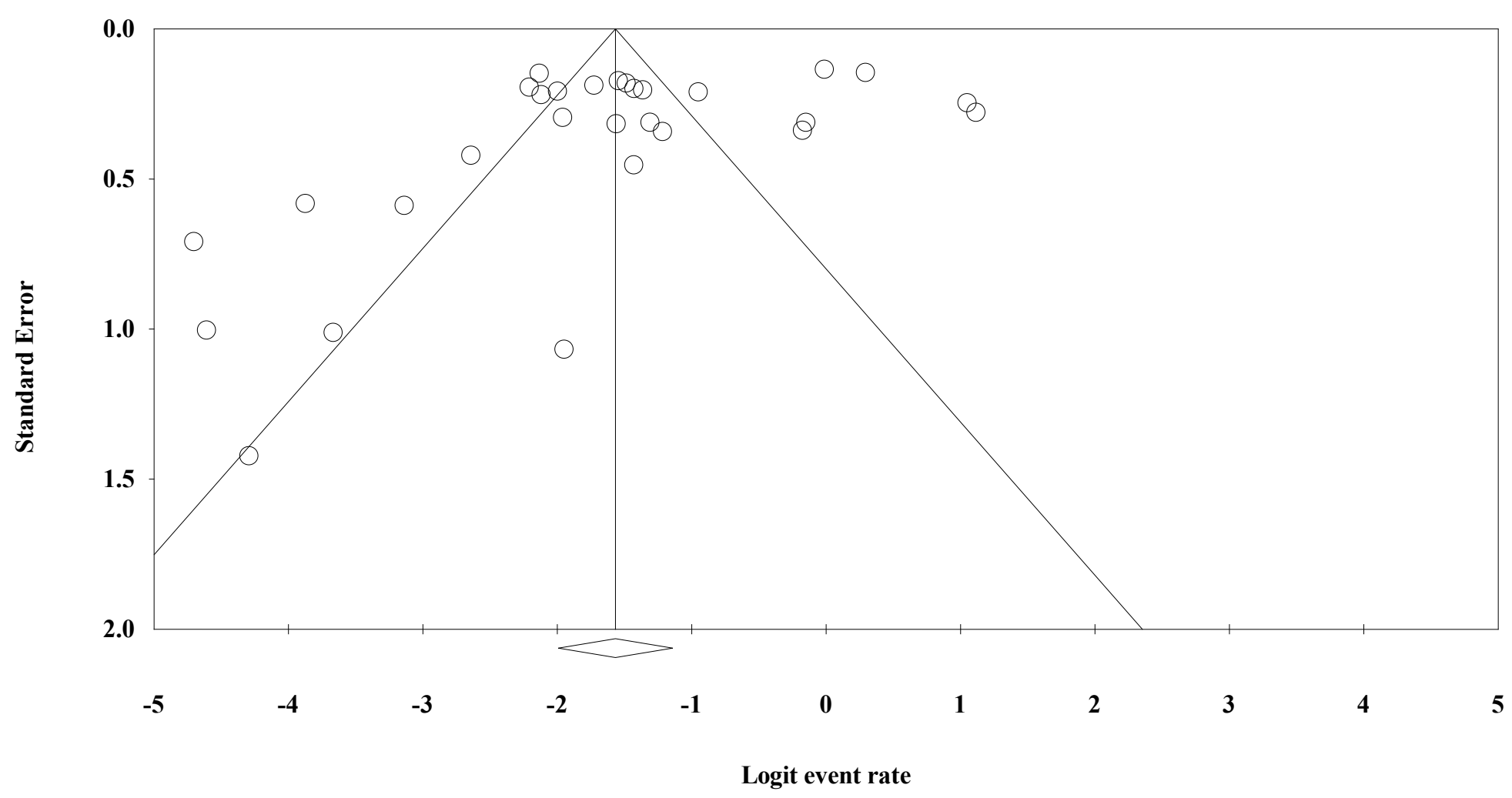

Supplementary Figure S13. Funnel plot used to assess small-study effects and potential publication bias among the datasets included in the meta-analysis. The horizontal axis represents the logit-transformed seroprevalence estimate, whereas the vertical axis represents the corresponding standard error. Egger's regression test showed no statistically significant funnel-plot asymmetry (intercept = −3.1108, SE = 1.7681, 95% CI: −6.7387 to 0.5171, $t = -1.759$, $df = 27$, $P = 0.090$). Duval and Tweedie's trim-and-fill procedure imputed no missing studies, and the adjusted random-effects pooled estimate remained unchanged at 17.3% (95% CI: 12.0–24.2%).
